# Rats exposed to *Alternaria* toxins in vivo exhibit altered liver activity highlighted by disruptions in riboflavin and acylcarnitine metabolism

**DOI:** 10.1007/s00204-024-03810-6

**Published:** 2024-06-28

**Authors:** Jesse T. Peach, Hannes Puntscher, Harald Höger, Doris Marko, Benedikt Warth

**Affiliations:** 1 Department of Food Chemistry and Toxicology, Faculty of Chemistry, University of Vienna, Vienna, Austria; 2 Center for Biomedical Research, Medical University of Vienna, Vienna, Austria; 3 Exposome Austria, Research Infrastructure and National EIRENE Node, Vienna, Austria

**Keywords:** Mycotoxins, *Alternaria*, Metabolomics, High-resolution mass spectrometry, Altertoxin II

## Abstract

Natural toxins produced by *Alternaria* fungi include the mycotoxins alternariol, tenuazonic acid and altertoxins I and II. Several of these toxins have shown high toxicity even at low levels including genotoxic, mutagenic, and estrogenic effects. However, the metabolic effects of toxin exposure from *Alternaria* are understudied, especially in the liver as a key target. To gain insight into the impact of *Alternaria* toxin exposure on the liver metabolome, rats (*n* = 21) were exposed to either (1) a complex culture extract with defined toxin profiles from *Alternaria alternata* (50 mg/kg body weight), (2) the isolated, highly genotoxic altertoxin-II (ATX-II) (0.7 mg/kg of body weight) or (3) a solvent control. The complex mixture contained a spectrum of *Alternaria* toxins including a controlled dose of ATX-II, matching the concentration of the isolated ATX-II. Liver samples were collected after 24 h and analyzed via liquid chromatography–high-resolution mass spectrometry (LC-HRMS). Authentic reference standards (> 100) were used to identify endogenous metabolites and exogenous compounds from the administered exposures in tandem with SWATH-acquired MS/MS data which was used for non-targeted analysis/screening. Screening for metabolites produced by *Alternaria* revealed several compounds solely isolated in the liver of rats exposed to the complex culture, confirming results from a previously performed targeted biomonitoring study. This included the altersetin and altercrasin A that were tentatively identified. An untargeted metabolomics analysis found upregulation of acylcarnitines in rats receiving the complex *Alternaria* extract as well as downregulation of riboflavin in rats exposed to both ATX-II and the complex mixture. Taken together, this work provides a mechanistic view of *Alternari* toxin exposure and new suspect screening insights into hardly characterized *Alternaria* toxins.

**Supplementary Information:**

The online version contains supplementary material available at 10.1007/s00204-024-03810-6.

## Introduction

Mycotoxins are secondary metabolites produced by certain mold fungi which, if exposed to humans or animals, can cause serious health consequences (Aichinger et al. 2021; Louro et al. 2024). Exposure to mycotoxins can occur through inhalation or dermal contact but is usually through the consumption of contaminated agricultural products (Barac 2019). Awareness of the risk presented by mycotoxin exposure has increased in tandem with the rising prevalence of not only acute health complications but also the development of a chronic health crisis (Wu et al. 2014; Claeys et al. 2020). As such, efforts have been made to increase regulatory measures and analytical identification methods (Chilaka et al. 2022). Yet, there are still major gaps in mycotoxin research and regulatory policy, highlighted by the need for increased research focused on several specific mycotoxin producing fungal genera, including *Alternaria* (Aichinger et al. 2021; Qin et al. 2022). The European Food Safety Authority has acknowledged these gaps and in 2016 concluded that exposure to *Alternaria* produced mycotoxins likely exceeds the threshold of toxicological concern and compound specific analyses of *Alternaria* mycotoxins should be conducted (Arcella et al. 2016).

*Alternaria*, generally visible as a black mold, is a major plant pathogen which infects cereal grains, fruit, and oil seeds, among others. *Alternaria* infection can be especially challenging for agricultural efforts as *Alternaria* can grow at low temperatures resulting in accumulation of toxins post-harvest in refrigerated conditions (Li et al. 2023). Food infested by *Alternaria* harbors a host of toxins including alternariol, alternariol monomethyl ether, tenuazonic acid and altertoxin I and II (Puntscher et al. 2018). Several of these toxins have been previously shown to have significant negative health impacts even at low levels. Mutagenic, genotoxic, cytotoxic, (anti-)estrogenic and immunomodulatory effects of isolated toxins and complex extracts have been shown in vitro and in vivo (Aichinger et al. 2019; Puntscher et al. 2019a, b; Gashgari et al. 2019; Hessel-Pras et al. 2019; Schmutz et al. 2019; Hohenbichler et al. 2020).

However, the impact of *Alternaria* toxin exposure has not been investigated at the metabolomics level in vivo. To fill this gap of knowledge, we performed an untargeted metabolomics analysis of liver samples from rats exposed to *Alternaria* toxins. This analysis is based on a prior study where the bioavailability, biotransformation, excretion, and toxicity of *Alternaria* toxins were investigated using a rat model (Puntscher et al. 2019a, b; Aichinger et al. 2022). These initial efforts showed genotoxic effects in vivo of both, isolated ATX-II and an *Alternaria*-produced toxin mixture designed to simulate the composition of a real-life exposure, although at higher levels than common exposures. Our analysis provides metabolic insights into the biological mechanisms of *Alternaria* toxin exposure by characterizing the endogenous rat metabolome and screening for *Alternaria* toxins and other small molecules leading to novel biomarker discovery.

## Methods

### Chemicals and reagents

LC–MS grade acetonitrile (ACN) and methanol  (MeOH) were obtained from Honeywell. LC–MS grade water was purchased from VWR. Formic acid was purchased from Promochem. Fully ^13^C-labeled dry yeast extract was procured from ISOtopic solutions (Vienna, Austria) and reconstituted in water per the manufactures' guidelines. Multi-analyte stock solutions of endogenous metabolites and exogenous toxicants (> 100) were prepared containing authentic standards purchased from Sigma and the Toronto Research Company (Supplemental Table 1).

### Animal experimental conditions

The in vivo study was conducted as previously described by Puntscher et al. (2019a) and was performed in accordance with the Austrian Animal Welfare Act 2012, BGBI. I Nr. 114/2012(TVG 2012). Briefly, 21 male Sprague Dawley^Ⓡ^ rats were procured and divided into a control group and two experimental groups. The control group received an innocuous sunflower supplement while the experimental groups received either isolated ATX-II toxin (0.7 mg/kg bw) or a complex extract of *Alternaria* toxins (50 mg/kg bw) with a matched ATX-II concentration, both in sunflower oil. Animals were harvested via heart punctuation 24 h post exposure at which time organs were immediately put on dry ice and then stored at − 80 °C until liquid chromatography–high-resolution mass spectrometry (LC–HRMS) analysis.

### Metabolite extraction

Rat liver samples stored at − 80 °C were allowed to gently thaw at − 20 °C overnight and then on ice until ready for excision. After samples were thawed, ~ 50 mg of material was removed using a scalpel and placed in a vial which was then placed on ice. Samples were kept on ice for the duration of the extraction whenever possible. The collected liver sample sections had a mean weight of 49.8 mg (0.5 mg RSD). Extraction was performed after the addition of 490 µL of MeOH:ACN:H2O (4:4:2). To this mixture 10 µL of isotopically labeled yeast extract and 250 mg of ceramic beads (~ 40 beads) were added after which the vial was placed in a tissue homogenizer (MP FastPreP-24 5G, MP Biomedicals). Using the homogenizer, samples were agitated at 6 m/s for 20 s twelve times with a 5-min rest between each cycle. Samples were then stored at − 20 °C overnight to aid protein precipitation.

After cold storage, samples were placed in a centrifuge at 18,000×*g* for 15 min. The supernatant was transferred to a new vial and placed in a vacuum concentrator at 4 °C until dryness and stored at − 80 °C until LC–HRMS analysis. Directly prior to analysis, dry samples were reconstituted with 200 µL of H2O:ACN (90:10), agitated via vortex three times for 30 s and sonicated for 5 min. Samples were spun in a centrifuge at 18,000×*g* for 5 min and finally placed in LC glass vials for analysis.

### LC–HRMS analysis

Rat liver extracts were analyzed on a SCIEX 7600 ZENO-TOF mass spectrometer coupled to an Agilent 1290 Infinity II UHPLC. Chromatographic separation was achieved using a Waters Acquity BEH-Amide HILIC column (2.1 mm × 100 mm, 1.7 µm) and a 15-min LC gradient. Water and ACN, both with 0.1% formic acid, were used as mobile phase A and B, respectively. The mobile phase gradient began at 99% B, which was held for 2 min before transitioning to 60% B over the course of 8 min. 60% B was held for 2 min before reverting to 99% B for the final 3 min. The column compartment was kept at 45 °C and the flow rate was kept constant at 0.4 mL/min. Ionization was performed via electrospray ionization in both positive and negative mode in two separate injections. Positive mode ionization was achieved with a spray voltage of 5500 V at 600 °C with a declustering potential at 80 V and a mass range of *m*/*z* 100–1000. Negative ionization was completed using a spray voltage of − 4500 V at 600 °C and the same declustering potential and mass range as used in the positive analysis. Fragmentation data were collected from pooled QC samples using the same conditions and the addition of a SWATH-acquisition experiment with mass windows between 50 and 100 *m*/*z*. Fragmentation was completed using a stepped collision energy design over the range of *m*/*z* 50–1000 which increased from 10 to 55 V with a CE spread of 5–10 V. This fragmentation method allowed for an almost universal precursor fragmentation data capture.

Additional analyses of pooled rat liver extracts, rat liver extracts spiked with the complex mixture, and the complex mixture itself were performed on the same analytical equipment with a different chromatographic column to aid in the annotation of specific less polar analytes with a Waters Acquity HSS-T3 (1.8 µm, 2.1 × 100 mm) reverse-phase column. A 20-min gradient was employed with 0.3 mM ammonium fluoride and ACN as mobiles phase A and B, respectively (Jamnik et al. 2022). The gradient began with 5%B which was held for 1 min before B was increased linearly to 18% at 1.8 min. B was again increased linearly to 35% at 4.2 min, 48% at 13 min and finally 90% at 15.8 min. From 17.7 to 20 min the column was flushed with 98% ACN. The column compartment was held at 40 °C and the flow rate was 0.4 mL/min. Data was collected in both positive and negative mode with SWATH acquisition as previously described.

### Data and statistical analysis

MS-DIAL was used to process data including peak picking, data mining, alignment, filtering, and normalization (Tsugawa et al. 2015). Features below an area of 250 were removed and a 0.01 Da window for MS and a 0.05 Da window for MS/MS data were used to align data. Feature datasets were adjusted by accounting for the variation in collected rat liver sample weight and via data-driven locally weighted scatterplot smoothing (LOWESS) normalization accompanied using isotopically labeled internal standards from labeled yeast amino acids spiked into each sample in MS-DIAL [13C-serine for (+) mode and 13C-arginine for (−) mode] to remove systemic sensitivity variation between samples. Fragmentation data collected using SWATH technology was explored using SIRIUS 4 software in combination with CSI:FingerID software and MS-DIAL along with a composite MS-DIAL spectral library for fragmentation matching to annotate features (Dührkop et al. 2015, 2019). An error window of 5 ppm was used for positive annotation along with an 80% spectral library match score. Authentic standard mixtures and 13C—labeled yeast, along with fragmentation data were also used to identify endogenous metabolites and xenobiotics (Supplemental Table 2). Identification levels were assigned to annotations based on confidence criteria proposed by Schymanski et al. (2014). Level 4 annotations relying solely on mass values were not considered while combinations of in silico, spectral library matching, 13C—labeled yeast and authentic reference standards were used to make composite level 1, 2 and 3 annotations. Statistical analysis was completed using MetaboAnalyst and several R packages including ggplot2 and in-house R scripts (Pang et al. 2022).

### Quality control

Isotopically labeled yeast were used as a system quality control measure. Fully labeled ^13^C-yeast were measured before and after positive and negative ionization analytical batches to ensure system reproducibility and stability. Fully labeled amino acids were identified and semi-quantitated. Based on this data coefficients of variation (CV) were determined, which indicated consistency throughout the analytical batch (Supplemental Table 3).

Quality control samples consisting of 100 µL of pooled reconstituted sample extract spiked with 10 µL of 13C yeast were also analyzed before, during and after the analytical batch. In total, five quality control injections were completed. As with the labeled isolated yeast, ^13^C-amino acids from the labeled yeast spiked into pooled rat liver extract samples were identified, semi-quantified and CV values were assessed. While not as consistent as the matrix-free labeled standards, the CV values still indicate reproducibility throughout the positive and negative ionization analytical batches (Supplemental Table 3). Annotated features were also compared in the pooled samples to ensure reproducibility and metabolites with a CV greater than 30% were removed from the dataset (Supplemental Table 4, Supplemental Fig. 1).

To account for potential background contamination, several blank samples were also generated. Solvent blanks were created using ACN and water taken from the same source as the dilution solvent and mobile phases. An additional extraction blank was created by processing an empty sample in parallel with the liver samples throughout the entirety of the extraction process. The solvent and extraction blank were used to determine systemic and method background noise. Features with abundances five times greater in the pooled rat liver extracts relative to the solvent and processing blanks were retained while features with a ratio under five were removed from the final dataset.

## Results

### Global untargeted analysis

**Fig. 1:**
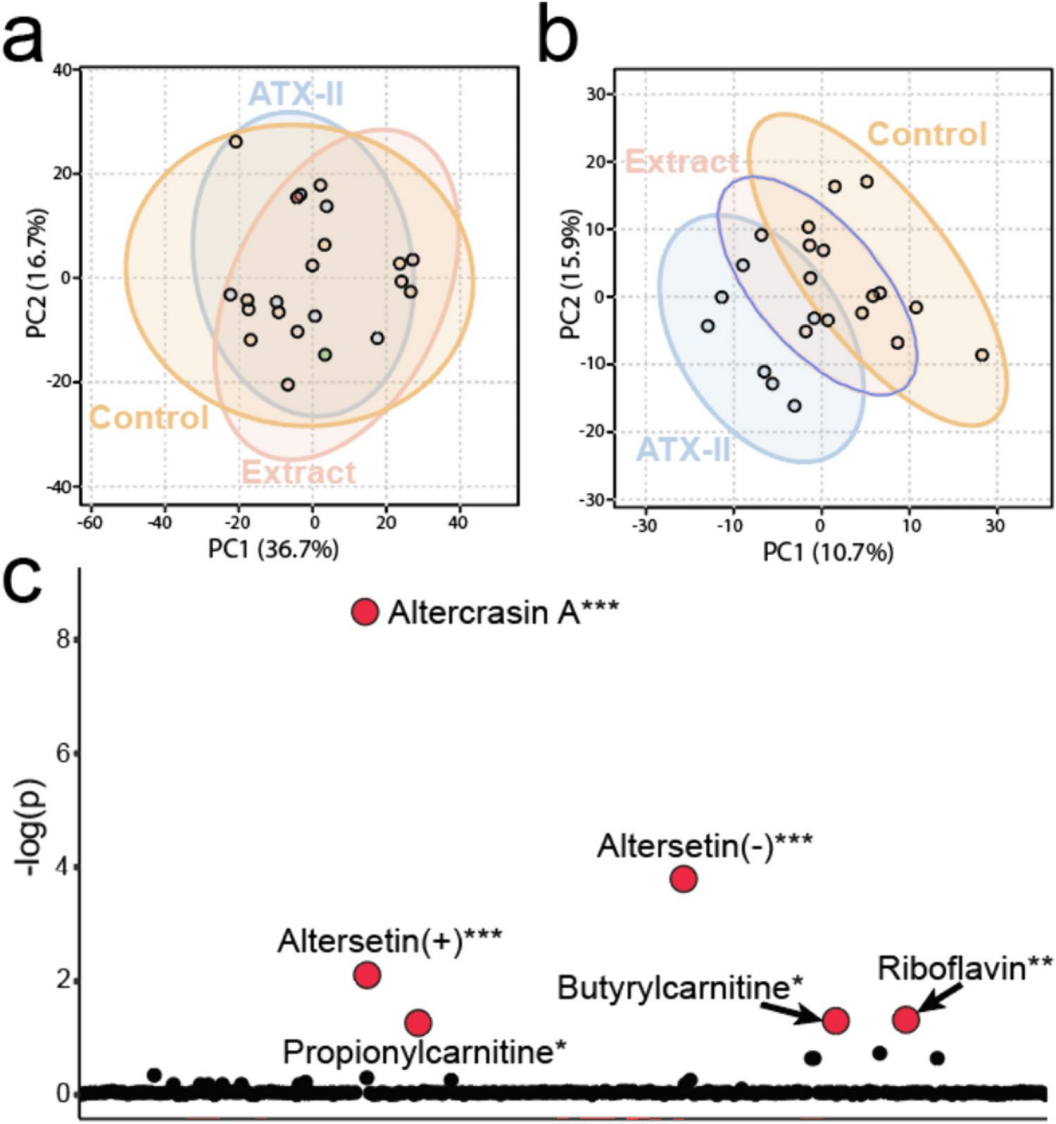
Untargeted analysis of LC–HRMS data. **a** 2D PCA of all three experimental groups in ESI (+) mode showing similar profiles indicating a well-balanced dose was applied in the experiment. **b** 2D PLS-DA of all three experimental groups in ESI (+) separation between the groups that can be elucidated. **c** Plot of FDR corrected ANOVA *P* values calculated for identified metabolites between the three experimental groups. Significant metabolites are indicated with red dots. Comparisons denoted with *** have a *P* value of < 0.01, ** have a *P* value of < 0.05 and * indicates a *P* value of < 0.1

This study was predicated on the administration of either an isolated mycotoxin, ATX-II, or a complex *Alternaria* toxin mixture with a profile simulating a real-life exposure scenario. To uncover the metabolic consequences of toxin exposure in our experimental groups, an untargeted analysis was undertaken with data from analytical batches collected in both ESI (+) and ESI (−) modes. The features were then curated and background noise from the analytical system and the extraction procedure were removed. CV values were calculated and features with CV values over 30% were removed. This resulted in 9,310 features in the ESI (+) dataset and 5798 in the ESI (−) dataset. A statistical analysis of metabolomic profiles indicated similar profiles between liver extracts from rats in the experimental groups, illustrated with the use of a 2-D principal component analysis (PCA) (Fig. 1a, Supplemental Fig. 2a). However, a supervised analysis using a 2-D partial least squares discriminating analysis revealed that some separation between the groups existed (Fig. 1b, Supplemental Fig. 2b). Interestingly, more separation was found between the ATX-II group and the control than between the cultured complex mixture and control group. The features providing this separation were elucidated using an ANOVA analysis which revealed 163 statistically significant features in the ESI (+) and 151 statistically significant features in the ESI (−) dataset which were significantly different between the experimental groups (*P* < 0.05) (Supplemental Table 5).

### Statistical analysis of annotated features

An ANOVA analysis of the annotated features elucidated several significantly different features (Supplemental Table 2). Figure 1c shows the annotated metabolites characterized in ESI (+) and (−) mode and their associated ANOVA *P* value. A total of five annotated metabolites were found to be at significantly different relative concentrations across the groups using a false discovery rate (FDR) corrected ANOVA. These significant metabolites included two acyl-carnitines, butyrylcarnitine and propionylcarnitine, riboflavin, altercrasin A and altersetin, which was isolated in both (+) and (−) mode and found to be significant in both.

### Metabolomics analysis of statistically significant metabolites

**Fig. 2:**
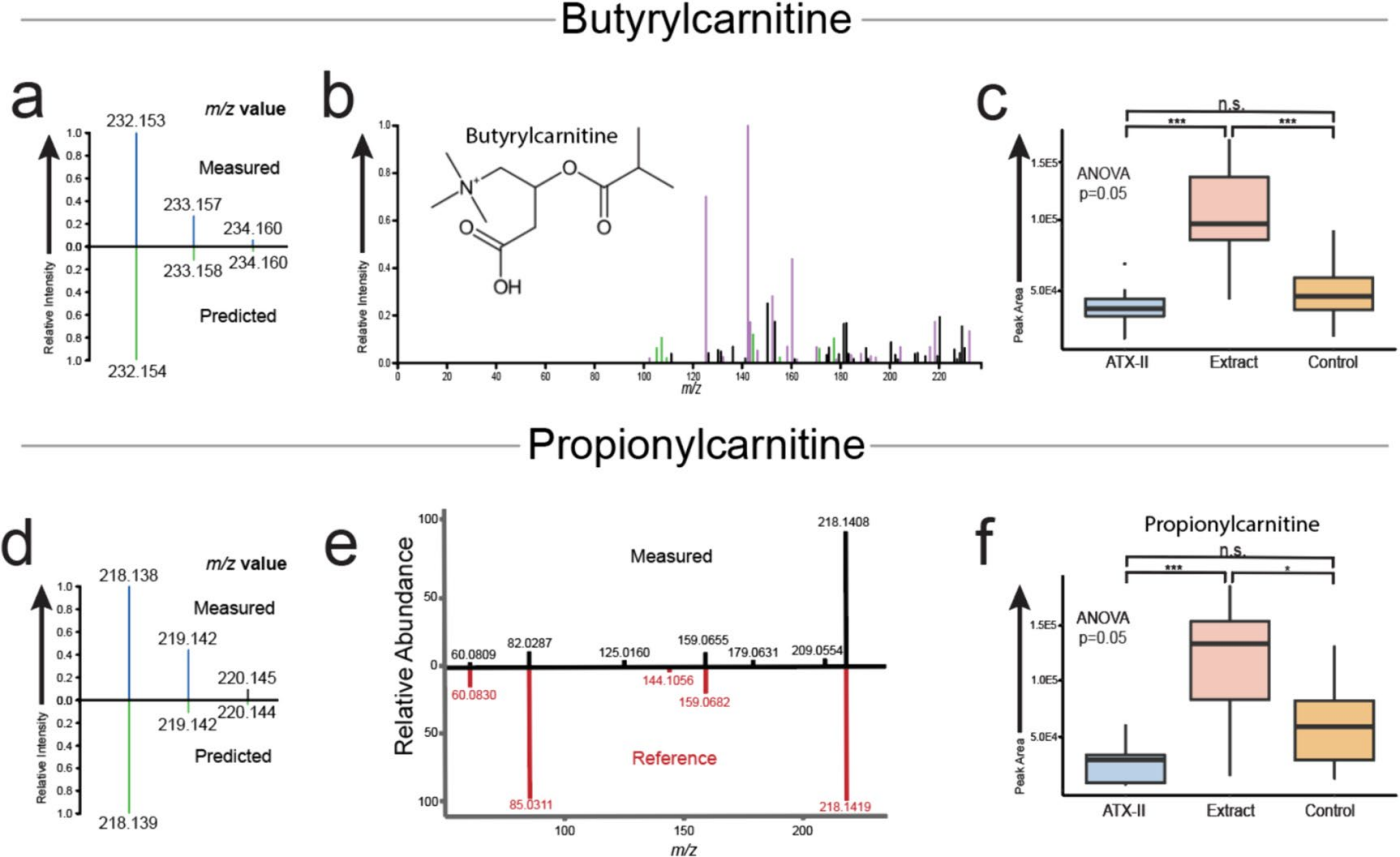
Analysis of acylcarnitines isolated in liver extracts. **a** Isotopic distribution of the acquired data in blue for the *m*/*z* value annotated as butyrylcarnitine and the predicted distribution in green. **b** In silico structural annotation for annotation of butyrylcarnitine using SIRIUS software with predicted structural fragments in purple and predicted formula fragments in green. **c** Plot of butyrylcarnitine relative concentrations found in each of the three experimental groups. Butyrylcarnitine concentrations were found to be significantly higher in complex toxin exposure group relative to both the ATX-II isolate and control group. **d** Isotopic distribution of the acquired data in blue for the *m*/*z* value annotated as propionylcarnitine and the predicted distribution in green. **e** Reference standard spectra comparison of propionylcarnitine vs measured spectra in rat livers annotation as propionylcarnitine. **f** Plot of propionylcarnitine relative concentrations showing a similar relationship as described in **a** for butyrylcarnitine. Comparisons denoted with *** have a *P* value of < 0.01, ** have a *P* value of < 0.05, * have a *P* value of < 0.1 and n.s. indicates a relationship that was found to be not significant using a *t* test

After isolating statistically important metabolites from the larger dataset, the individual metabolites were examined in closer detail to assess data quality and their statistical relationship between the experimental groups. Beginning with acylcarnitines, data quality was analyzed in the QC samples which demonstrated robust reproducibility (Supplemental Table 4, Supplemental Fig. 1). Although butyrylcarnitine was annotated using in silico methods, an authentic reference standard was used to identify propionylcarnitine, lending confidence to the annotation of both acylcarnitines (Fig. 2a, b, d, e). After ensuring quality, reproducible data, the relative concentrations were analyzed by experimental group (Fig. 2c, f). One of the statistically important acylcarnitines, butyrylcarnitine, was significantly upregulated in the liver extracts from rats receiving the *Alternaria* complex culture. This trend was calculated to be significant using a t test between the *Alternaria* complex mixture liver extracts and the ATX-II isolate liver extracts as well as between the *Alternaria* complex mixture liver extracts and the control liver extracts (*P* < 0.01). There was no significant difference between the ATX-II isolate and control samples. Propionylcarnitine exhibited similar metabolic regulation and was significantly increased in the *Alternaria* complex mixture relative to the ATX-II isolate samples. Although samples from rats receiving the *Alternaria* complex culture indicated an increase when compared to the control samples, the increase was statistically weak (*P* value 0.068).

**Fig. 3:**
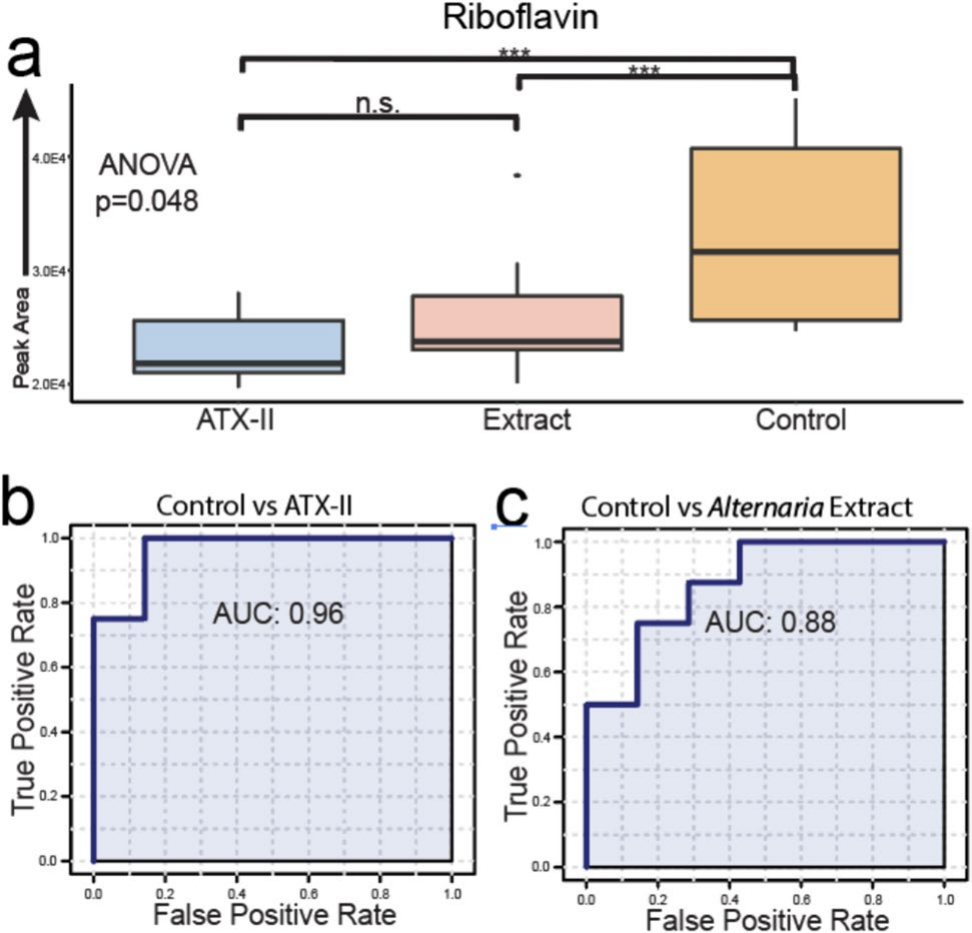
Statistical analysis of riboflavin levels. **a** Plot of relative concentrations of riboflavin by experimental group in rat liver samples extracts. Riboflavin was significantly downregulated in both the complex mixture exposure group and the ATX-II isolate relative to the control. Comparisons marked by *** have a *P* value of > 0.01 and comparisons with a n.s. were found to be not significant. **b**, **c** AUC measurements for ROC biomarker analyses in comparisons between **b** control and ATX-II isolate experimental groups and **c** control and the complex toxin mixture experimental groups. Both biomarker analyses have very high AUCs indicative of low false positive and negative values allowing for high predictive probability making riboflavin an excellent biomarker for *Alternaria* and ATX-II exposure

Another metabolite that was statistically significantly different between experimental groups was riboflavin. Riboflavin was initially annotated by a spectral library match during the untargeted analysis and subsequently identified using a reference standard in a follow-up targeted analysis after purchasing an authentic reference standard. Hence, it represents a confident level 1 identification (Supplemental Fig. 3). Applying a data quality analysis similar to that for the acylcarnitines indicated excellent reproducibility for riboflavin in the QC samples with a calculated CV value of 0.47% (Supplemental Table 4). Riboflavin relative concentrations were investigated between the experimental groups and a strong pattern emerged (Fig. 3a). Riboflavin was calculated to be significantly downregulated in both the *Alternaria* mixture and the ATX-II isolate groups. There was no significant difference between the *Alternaria* and ATX-II groups. As riboflavin was found to be associated with similar effects in both mycotoxin-containing groups, it was further examined as a biomarker for *Alternaria* and ATX-II exposure. A receiver operator characteristic analysis was completed to determine if riboflavin levels could be used to predict both the complex extract exposure as well as ATX-II isolate exposure (Fig. 3b, c). The area under the curve (AUC) values calculated for both analyses were excellent. Riboflavin was slightly more predictive of ATX-II exposure with an AUC of 0.96 relative to the AUC value of 0.88 found for *Alternaria* culture mixture exposure.

### Metabolic pathway analysis

**Fig. 4:**
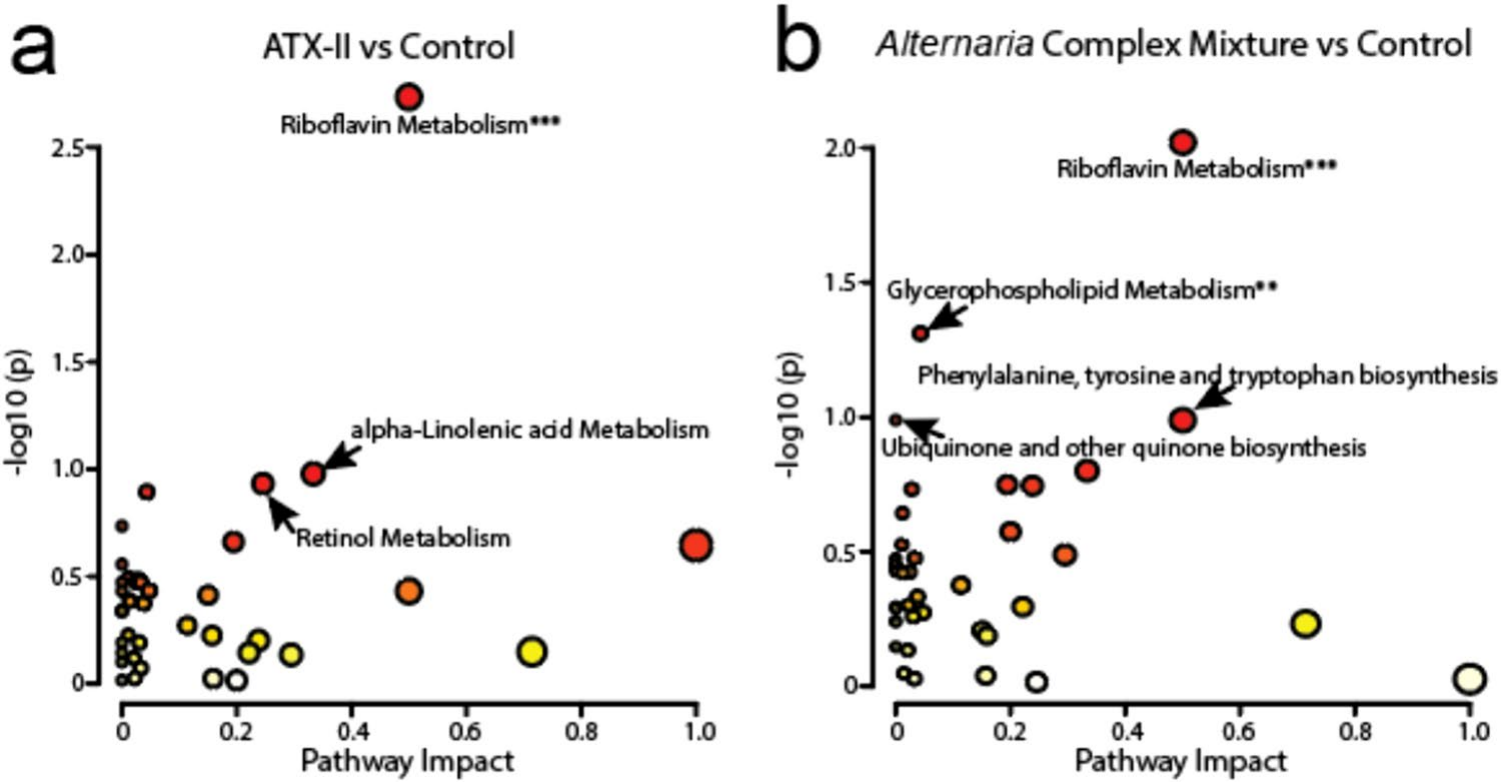
Pathway analysis of identified metabolites. **a** ATX-II isolate and control groups comparative analysis indicating riboflavin metabolism as a significantly disrupted pathway. **b** Complex toxin mixture exposure and control groups comparative analysis showing that riboflavin metabolism is significantly disrupted. Significance and pathway impact are plotted on the *y*- and *x*-axis, respectively. The size of the dot is indicative of the pathway impact while red colors indicate higher levels of significance. Pathways marked with an * have *P* values < 0.1, ** denoted pathways have *P* values < 0.05 and *** indicated pathways have *P* values > 0.01

An analysis of metabolic pathways impacted by the different *Alternaria*-based exposures was also completed using the full datasets and the pathway analysis function in MetaboAnalyst. A comparison of the ATX-II isolate and the control liver extracts indicated significant differences in riboflavin metabolism (Fig. 4a). Other pathways dysregulated in this comparison included alpha-linoleic acid and retinol metabolism. A comparison of *Alternaria* extract and control samples again indicated a significant disruption in riboflavin metabolism (Fig. 4b). Several other pathways were found to be dysregulated including glycerophospholipid metabolism, which was found to be significantly dysregulated. Other pathways such as phenylalanine, tyrosine and tryptophan biosynthesis and ubiquinone and other quinone biosynthesis were altered, although they were not as significant. A final pathway analysis of *Alternaria* complex mixture and ATX-II isolate samples revealed valine, leucine and isoleucine biosynthesis dysregulation as well as moderate effects to retinol metabolism and porphyrin metabolism (Supplemental Fig. 4).

### *Alternaria* toxin exposomics screening

**Fig. 5:**
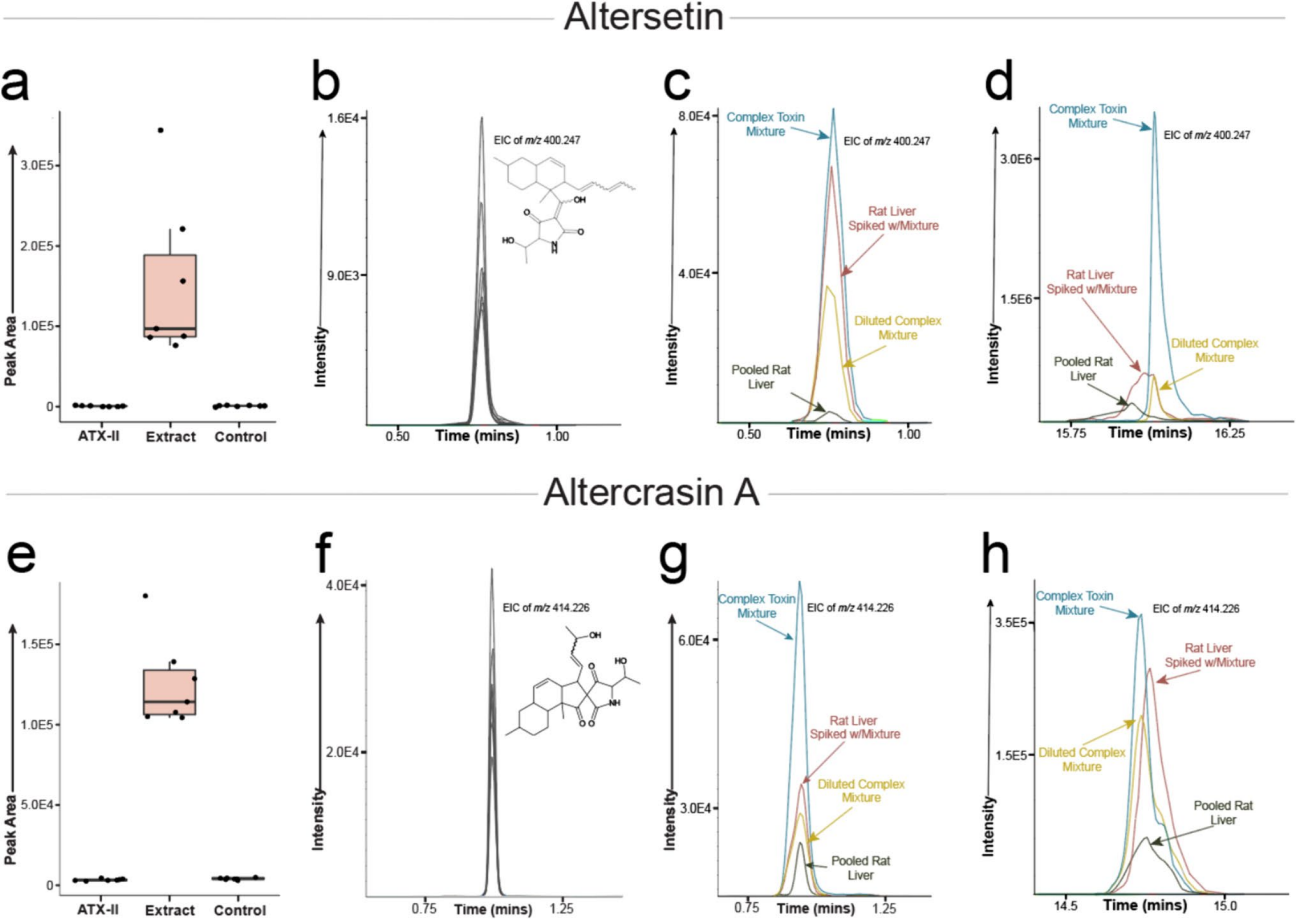
*Alternaria*-specific metabolite levels. **a** Relative concentrations of altersetin by experimental group, isolated solely in livers from rats exposed to the complex toxin mixture (“Extract”). **b**–**d** EICs of *m*/*z* 400.247, the value associated with altersetin, for **b** individual rat liver extracts from rats exposed to the complex mixture analyzed using the HILIC method, **c** pooled liver extracts from rats exposed to the complex mixture and the complex mixture analyzed using the HILIC method and **d** pooled rat liver extracts from rats receiving the complex mixture and the complex mixture analyzed using the reverse-phase method. **e** Relative concentrations of altercrasin A by experimental group, isolated solely in livers from rats exposed to the complex toxin mixture (“Extract”). **f**–**h** EICs of *m*/*z* 414.226, the value associated with altercrasin A, for **f** individual liver extracts from rats receiving the complex mixture analyzed using the HILIC method, **g** pooled liver extracts from rats exposed to the complex mixture and the complex mixture analyzed using the HILIC method and **h** pooled liver extracts from rats receiving the complex mixture and the complex mixture analyzed using the reverse-phase method

To determine if *Alternaria* produced toxins persisted in the liver after 24 h, an investigation into the presence of known *Alternaria* mycotoxins was conducted. Although concentrations have been previously reported in biological matrices, to our knowledge *Alternaria* toxins and metabolites have not been explored in liver samples. Commonly reported *Alternaria*-produced toxins and metabolites were screened for including alternariol, alternariol monomethyl ether, tenuazonic acid ATX-II and ATX-I. Neither ATX-I nor ATX-II was determined in either the isolate or the complex mixture exposed liver extracts, which was consistent with previous results in rat plasma and urine (Puntscher et al. 2019a, b). A further screening for mycotoxins yielded the presence of altersetin, a toxin with antibiotic properties produced by *Alternaria*, which was found exclusively in the rats receiving the complex mixture (Fig. 5a, b) (Hellwig et al. 2002). Altersetin was annotated using both spectral library matching and in silico predictive modeling and had a CV of 2.5% in pooled QC samples, indicating low technical variability (Supplemental Fig. 5). An analysis of pooled samples from rats receiving the complex mixture and the mixture itself revealed the presence of altersetin in the complex mixture with the same *m*/*z* and retention time found in the rat liver extracts (Fig. 5c). Additional reversed-phase LC–HRMS analysis, found that altersetin had a retention time of 16 min and showed the same pattern with respect to pooled rat samples and the complex mixture as with the initial HILIC analytical analysis (Fig. 5d).

Although most common *Alternaria* produced toxins were screened for and not detected in liver samples after 24 h, a deep exploration of the data via non-targeted analysis revealed the potential presence of the toxin altercrasin A, which was isolated using an in silico analysis and only found in the rats receiving the complex mixture (Fig. 5e, f). Since neither an authentic reference standard nor an MS/MS library entry is available for this toxin to the best of our knowledge, the annotation is assigned a confidence level of 3 (Supplemental Fig. 6).

To further investigate this potentially interesting finding, the complex mixture from the cultured extract was examined independently using the same LC–HRMS method and the *m*/*z* associated with altercrasin A was found at the same retention time as in the rat liver extracts (Fig. 5g). This demonstrates that the annotated feature likely originates from the extract. To investigate this peak of interest further, a sample of the liver extract and the cultured extract were subjected to complementary reversed phase LC–HRMS and, as with the HILIC analysis, the *m*/*z* for altercrasin A and retention times between the sample types matched in both, the rat liver extracts and the complex mixture administered during the experiment (Fig. 5h). The analysis of altercrasin A revealed good peak shape in an extracted ion chromatogram of the MS1 data and MS/MS collected data indicated a matching isotopic distribution as well as promising structural fragmentation matching annotation (Supplemental Fig. 6a, b). Technical variability in the pooled QC samples was examined and a CV value of 12% was calculated for the m/z value associated with altercrasin A (Supplemental Table 4).

## Discussion

This study investigated the in vivo metabolic effects of *Alternaria* toxin exposure in a rat model, building on analysis from the same animal experiments which elucidated important insights into the absorption, metabolism and excretion of *Alternaria* toxins, especially ATX-II (Puntscher et al. 2019a, b). Toxins were found to be greatly reduced in concentration after three hours while some toxins were still isolated at very low levels in some biological matrices after 24 h. Additionally, the genotoxicity effects of *Alternaria*, and specifically ATX-II, were described in vivo (Aichinger et al. 2022). Genotoxicity was determined in the colon of the rats receiving an *Alternaria* complex mixture and those receiving an ATX-II isolate. By comparison, an analysis of liver samples showed little genotoxic effects. Surprisingly, the ATX-II isolate demonstrated a higher genotoxic effect in the colon relative to the mycotoxin mixture, indicating a potential source of mycotoxin metabolism or protection activation provided by components of the complex mixture as it contained the same concentration of ATX-II. Although these studies expanded the understanding of *Alternaria* exposures, gaps remained. By harnessing LC–HRMS technology, we were able to investigate the effects of exposure in vivo and determine dysregulated metabolites and metabolic pathways as well as isolate a biomarker for *Alternaria* exposure. Our analysis indicated a complex metabolic shift associated with *Alternaria* exposure highlighted by the persistent presence of the *Alternaria* produced mycotoxin altercrasin A and metabolite altersetin isolated from *Alternaria* exposed rat livers 24 h post-exposure as well as an increase in acyl-carnitines in rats exposed to an *Alternaria* mycotoxin mixture and the downregulation of riboflavin levels in livers from rats exposed to either the *Alternaria* complex mixture or an isolated *Alternaria* mycotoxin, ATX-II.

An interesting component to earlier studies was the finding that ATX-II exposed rat colons exhibited higher genotoxic damage markers than the complex mixture (Aichinger et al. 2022). Although genotoxic markers were not seen in the liver, a similar result was found with respect to the global untargeted data analysis. A supervised approach to determine the variation between each experimental group indicated that the liver samples from rats exposed to the *Alternaria* mycotoxin mixture were more similar to the control than the ATX-II isolate (Fig. 1b, Supplemental Fig. 2b). These results, taken with conclusions reached concerning the toxicity protection offered in the rat colon from the complex mixture relative to the ATX-II isolate demonstration in the previous study, seem to point to a mixture effect in the *Alternaria* cultured complex mixture. This could be, as previously conjectured, an increase in the production or release of a metabolite with a protective or mediating function or the activation of an enzyme capable of metabolizing toxins produced by *Alternaria*.

Although liver samples did not show genotoxic effects before, our metabolomics analysis indicated several differences in liver samples 24-h post-exposure. An important finding is that of the toxins altersetin and altercrasin A in the liver samples from rats exposed to the *Alternaria* mycotoxin mixture (Fig. 5a and e). Little is known about altercrasin A, a decalin derivative produced by *Alternaria*, yet its persistent presence in the liver is notable and further research should be completed (Yamada et al. 2019; Jiang et al. 2020). As mycotoxins have been shown to negatively impact liver metabolism and health, this could be an important component for acute and chronic *Alternaria* exposure risks (Hasuda et al. 2022).

Along with altercrasin A, short chain acyl-carnitines were calculated via a statistical analysis to be upregulated in livers from rats exposed to the *Alternaria* cultured mixture. Specifically, the acylcarnitines butyrylcarnitine and propionylcarnitine demonstrated elevated levels in the complex mixture samples relative to the control and ATX-II isolate samples (Fig. 2c, f). Acylcarnitines are esters of L-carnitine and fatty acids which transport fatty acids across the mitochondrial membrane and initiate the β-oxidation pathway, making them integral to energy metabolism. Acylcarnitines are, therefore, vital to energy metabolism and are often used as biomarkers for mitochondrial activity and to screen for disorders effecting β-oxidation (McCann et al. 2021). Recently, the use of acyl-carnitines as markers for disease states has expanded as acylcarnitines have been posited as biomarkers for a wide range of diseases including diabetes, cancers and cardiovascular disease (Dambrova et al. 2022).

The liver, specifically, has been shown to be a major systemic contributor of not just acylcarnitines, but short chain acylcarnitines such as butyrylcarnitine (Xu et al. 2016). Elevated levels of short chain acyl-carnitines have also been reported to be associated with increased inflammation and disease states in the liver (Ghosh et al. 2016; Li et al. 2019). Taken together, it is likely that a component of the complex mycotoxin mixture is still impacting liver energy metabolism 24 h after exposure. Given the results from our *Alternaria* compound screening analysis focused on mycotoxins, this increase in acylcarnitines could be due to effects from a persistent component of the complex toxin mixture.

Riboflavin was also shown, like acylcarnitines, to be differentially regulated in our experimental groups through our metabolomics analysis (Fig. 3a). *Alternaria* exposure, either via the cultured *Alternaria* or the ATX-II isolate exposure led to significant downregulation in riboflavin concentrations. The degree of downregulation was similar between both routes of exposure indicating ATX-II alone impacts riboflavin metabolism. The variation in riboflavin levels between the groups receiving either of the *Alternaria* exposures was statistically robust enough that riboflavin was found to have strong statistical value as a biomarker and predictor of exposure (Fig. 3b, c).

Riboflavin, or vitamin B2, is an essential nutrient which plays vital roles in several metabolic pathways such as central carbon energy metabolism as the precursor for both flavin adenine dinucleotide (FAD) and flavin mononucleotide (FMN) and stress responses including exhibiting anti-inflammatory effects (Ashoori and Saedisomeolia 2014; Barile et al. 2016; Ahn and Lee 2020). Human and animal model studies have demonstrated the negative impacts of riboflavin deficiency which include growth retardation, fatigue, depression, lipid accumulation and nutrient absorption (Thakur et al. 2017; Mosegaard et al. 2020). For example, decreased riboflavin concentrations have been shown to limit iron uptake in the small intestine leading to anemia in human populations (Aljaadi et al. 2022). Liver specific pathologies have also been attributed to riboflavin deficiency including the development of fatty liver and liver enlargement. Tang et al. (2022) demonstrated that certain liver pathologies were regulated by specific proteins which were associated with riboflavin deficiency. Proteins integral to fatty acid β-oxidation and mitochondrial electron transport chain function were decreased while triacylglycerol and cholesterol biosynthesis were increased. Ultimately, these results provide a mechanistic avenue for the development of liver metabolic disorders arising from riboflavin deficiency.

**Fig. 6:**
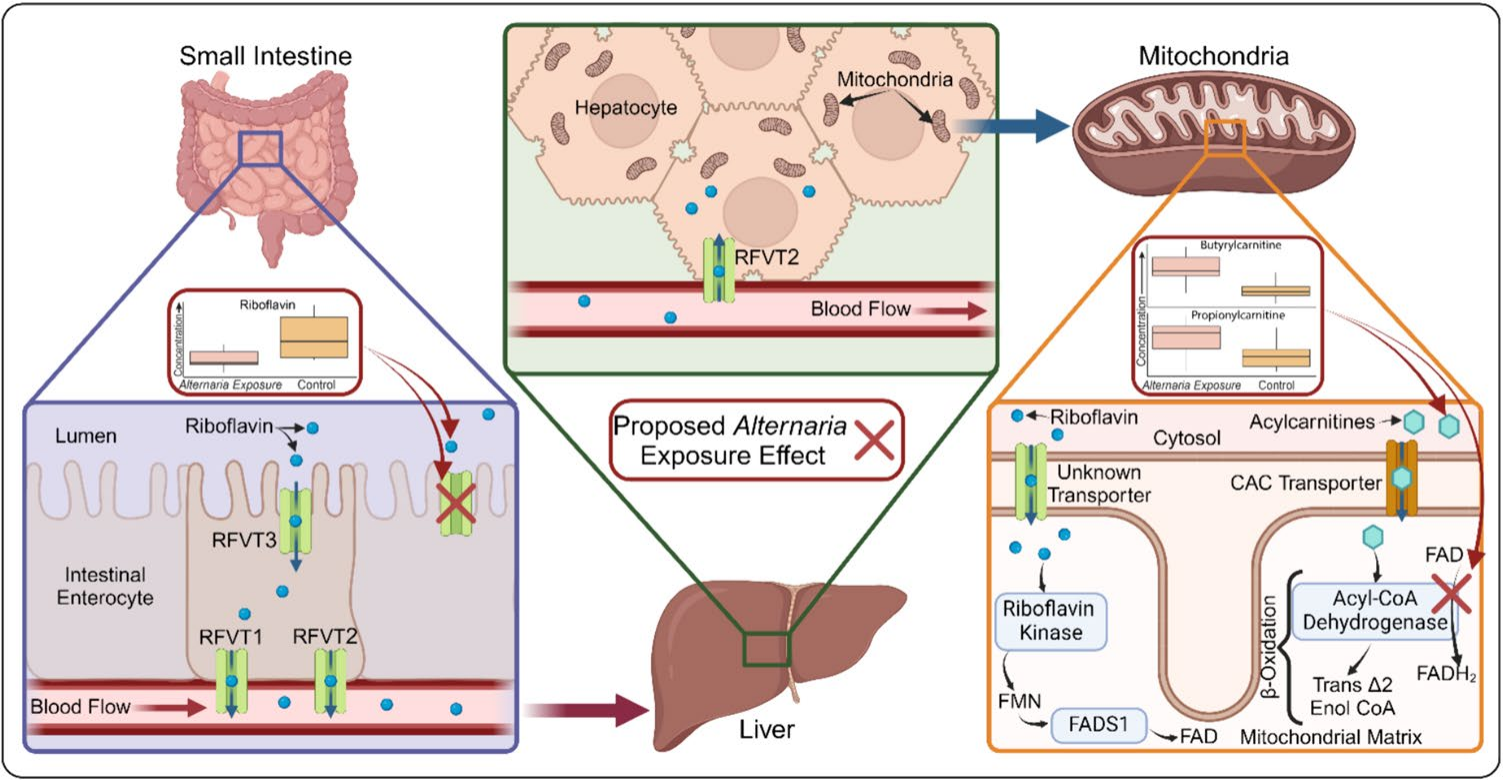
Riboflavin metabolism and *Alternaria* toxin action. The journey of riboflavin begins with absorption in the gut followed by transport to the liver and, ultimately, the mitochondria. The role of riboflavin in β-oxidation is also shown as well as integrated data collected from our study indicating riboflavin and acylcarnitine level disparities between experimental groups. Red X marks proposed disruptions in the pathway arising from *Alternaria* toxin exposure and include limited riboflavin uptake as well as β-oxidation suppression stemming from inhibition of Acyl-CoA Dehydrogenase due to a lack of essential FAD

Riboflavin deficiency can be problematic as riboflavin is not endogenously produced in animals and humans and is therefore recovered solely from dietary sources such as dairy and meat products, where it is prevalent (Suwannasom et al. 2020). Since riboflavin needs to be acquired via dietary measures, absorption rates in the intestinal tract will greatly influence systemic and tissue specific levels (Levy and Jusko 1966). Riboflavin is transported from the gut by a group of solute carrier transporters (SLC) type transporters. Characterized riboflavin transporters include RFVT1, RFVT2 and RFVT3 and are integral to the absorption of riboflavin, which is thought to occur mostly in the small intestine where RFVT1 is expressed on the apical side and RFVT2 and RFVT3 are expressed on the basal side (Yonezawa and Inui 2013; Udhayabanu et al. 2017) (Fig. 6). It is likely, given our data, that toxins produced by *Alternaria* alter this pathway at some point leading to a decrease in riboflavin uptake across the epithelial cell layer in the intestinal tract. This has been demonstrated previously with the mycotoxin deoxynivalenol (DON) which was shown to limit uptake of glucose, 5-methyltetrafolic acid, molybdenum, and magnesium in the intestinal tract (Pinton and Oswald 2014). These results are also in line with the conclusions from previous *Alternaria* toxin exposure results in rat models where the gastrointestinal tract was impacted by both the ATX-II isolate and a complex *Alternaria* toxin mixture (Aichinger et al. 2022).

Once across the intestinal epithelium, riboflavin carrier proteins (RCP) transport riboflavin via blood to targeted areas of the body, including the liver where small amounts of riboflavin are stored. Riboflavin shuttled to the liver enters hepatocytes through RFVT2 transporters and is then transported across the mitochondrial membrane through an unknown mechanism (Yao et al. 2013). Once in the mitochondrial matrix, riboflavin is catabolized by riboflavin kinase to form FMN. FMN can be further modified by the activity of FAD synthetase to generate FAD, a required cofactor in β-oxidation.

β-Oxidation, or the catabolism of fatty acids, only occurs in the mitochondria which presents challenges due to the negative charges associated with fatty acids. This makes crossing the mitochondrial membrane difficult and requires a transporter and modifications to make possible. The journey of fatty acids to the mitochondria begins in the cytosol, where fatty acyl-CoA synthetase forms a thioester bond between fatty acids and coenzyme A, creating an acyl-CoA (Watkins 2013). Following the synthesis of acyl-CoA, carnitine acetyltransferase activates transfer of the acyl group to carnitine to create acylcarnitines. Acylcarnitines are then shuttles across the membrane via the carnitine acylcarnitine carrier (CAC) (Tonazzi et al. 2013) (Fig. 6). Once in the mitochondrial matrix, the carnitine is removed and replaced by coenzyme A. The resulting fatty acyl-CoA then enters the β-oxidation cycle. Each cycle of β-oxidation cleaves two carbons, released as acetyl-CoA and two electron carriers, NADH and FADH2. The first step in the process requires the reduction of FAD to FADH2 and without FAD, β-oxidation will not occur (Swigoňová et al. 2009). In this way, FAD regulates β-oxidation and the conversion of acylcarnitines to acyl-CoA (Adeva-Andany et al. 2019). Since FAD production in hepatocytes is influenced by riboflavin transport to the liver and across the intestinal epithelium, decreased riboflavin uptake would lead to a decrease in β-oxidation and an increase in acyl-carnitines. This result is seen in the rat livers exposed to the complex toxin mixture but not in rat livers exposed to solely ATX-II. A component in the *Alternaria* complex mixture, possibly altercrasin A, appears to be decreasing FAD concentrations in the mitochondria, leading to the inability to start the conversion of acyl-carnitines in β-oxidation. This could be accomplished by limiting riboflavin transport into the mitochondrial matrix or by inhibiting riboflavin conversion to FMN or FAD or by disrupting β-oxidation itself, compounding the initial decrease in riboflavin concentrations likely due to decreased intestinal absorption.

*Alternaria* and their associated toxins represent an underestimated health risk which has been compounded by a lack of information concerning toxin metabolism and their systematic investigation of effects on the metabolome. Previous research has shown *Alternaria* to be a potent toxin producer with genotoxic activity. Here we present additional findings indicating that *Alternaria* exposure results in an accumulation of short chain acylcarnitines in the liver as well as likely leading to a decrease in riboflavin absorption in the intestinal tract translating to a decrease in liver concentrations. These physiological changes would limit β-oxidation resulting in decreased energy production while at the same time reducing the availability of a functional vitamin like riboflavin and the associated coenzymes FAD and FMN, causing negative health consequences. These results, taken with previous work indicating the genotoxicity of certain *Alternaria* toxins, indicate the importance of further health-based investigations and the potential need for regulations regarding *Alternaria* toxins in food and animal feed.

## Supplementary Information

Below is the link to the electronic supplementary material.

Supplementary file1 (DOCX 3339 KB)

## Data Availability

The LC-HRMS data is publicly available at https://www.metabolomicsworkbench.org. Any additional requests for data access should be directed to the corresponding author.

## References

[CR1] Adeva-Andany MM, Carneiro-Freire N, Seco-Filgueira M, Fernández-Fernández C, Mouriño-Bayolo D (2019) Mitochondrial β-oxidation of saturated fatty acids in humans. Mitochondrion 46:73–90. 10.1016/j.mito.2018.02.009 29551309 10.1016/j.mito.2018.02.009

[CR2] Ahn H, Lee G-S (2020) Riboflavin, vitamin B2, attenuates NLRP3, NLRC4, AIM2, and non-canonical inflammasomes by the inhibition of caspase-1 activity. Sci Rep 10:19091. 10.1038/s41598-020-76251-7 33154451 10.1038/s41598-020-76251-7 PMC7645791

[CR3] Aichinger G, Krüger F, Puntscher H, Preindl K, Warth B, Marko D (2019) Naturally occurring mixtures of *Alternaria* toxins: anti-estrogenic and genotoxic effects in vitro. Arch Toxicol 93:3021–3031. 10.1007/s00204-019-02545-z 31559443 10.1007/s00204-019-02545-z

[CR4] Aichinger G, Del Favero G, Warth B, Marko D (2021) *Alternaria* toxins—still emerging? Compr Rev Food Sci Food Saf 20:4390–4406. 10.1111/1541-4337.12803 34323368 10.1111/1541-4337.12803

[CR5] Aichinger G, Pahlke G, Puntscher H, Groestlinger J, Grabher S, Braun D, Tillmann K, Plasenzotti R, Del Favero G, Warth B, Höger H, Marko D (2022) Markers for DNA damage are induced in the rat colon by the *Alternaria* toxin altertoxin-II, but not a complex extract of cultured *Alternaria alternata*. Front Toxicol 4. 10.3389/ftow.2022.977147 10.3389/ftox.2022.977147 PMC9638006 36353200

[CR6] Aljaadi AM, Devlin AM, Green TJ (2022) Riboflavin intake and status and relationship to anemia. Nutr Rev 81:114–132. 10.1093/nutrit/nuac043 36018769 10.1093/nutrit/nuac043

[CR7] Arcella D, Eskola M, Gómez Ruiz JA (2016) Dietary exposure assessment to *Alternaria* toxins in the European population. EFSA J 14:e04654. 10.2903/j.efsa.2016.4654 10.2903/j.efsa.2016.4654 PMC13088870 42006026

[CR8] Ashoori M, Saedisomeolia A (2014) Riboflavin (vitamin B_2_) and oxidative stress: a review. Br J Nutr 111:1985–1991. 10.1017/S0007114514000178 24650639 10.1017/S0007114514000178

[CR9] Barac A (2019) Mycotoxins and human disease. In: Presterl E (ed) Clinically relevant mycoses: a practical approach. Springer International Publishing, Cham, pp 213–225. 10.1007/978-3-319-92300-0_14

[CR10] Barile M, Giancaspero TA, Leone P, Galluccio M, Indiveri C (2016) Riboflavin transport and metabolism in humans. J Inherit Metab Dis 39:545–557. 10.1007/s10545-016-9950-0 27271694 10.1007/s10545-016-9950-0

[CR11] Chilaka CA, Obidiegwu JE, Chilaka AC, Atanda OO, Mally A (2022) Mycotoxin regulatory status in Africa: a decade of weak institutional efforts. Toxins 14:442. 10.3390/toxins14070442 35878180 10.3390/toxins14070442 PMC9321388

[CR12] Claeys L, Romano C, De Ruyck K, Wilson H, Fervers B, Korenjak M, Zavadil J, Gunter MJ, De Saeger S, De Boevre M, Huybrechts I (2020) Mycotoxin exposure and human cancer risk: a systematic review of epidemiological studies. Compr Rev Food Sci Food Saf 19:1449–1464. 10.1111/1541-4337.12567 33337079 10.1111/1541-4337.12567

[CR13] Dambrova M, Makrecka-Kuka M, Kuka J, Vilskersts R, Nordberg D, Attwood MM, Smesny S, Sen ZD, Guo AC, Oler E, Tian S, Zheng J, Wishart DS, Liepinsh E, Schiöth HB (2022) Acylcarnitines: nomenclature, biomarkers, therapeutic potential, drug targets, and clinical trials. Pharmacol Rev 74:506–551. 10.1124/pharmrev.121.000408 35710135 10.1124/pharmrev.121.000408

[CR14] Dührkop K, Shen H, Meusel M, Rousu J, Böcker S (2015) Searching molecular structure databases with tandem mass spectra using CSI:FingerID. Proc Natl Acad Sci 112:12580–12585. 10.1073/pnas.1509788112 26392543 10.1073/pnas.1509788112 PMC4611636

[CR15] Dührkop K, Fleischauer M, Ludwig M, Aksenov AA, Melnik AV, Meusel M, Dorrestein PC, Rousu J, Böcker S (2019) SIRIUS 4: a rapid tool for turning tandem mass spectra into metabolite structure information. Nat Methods 16:299–302. 10.1038/s41592-019-0344-8 30886413 10.1038/s41592-019-0344-8

[CR16] Gashgari R, Ameen F, Al-Homaidi E, Gherbawy Y, Al Nadhari S, Vijayan V (2019) Mycotoxigenic fungi contaminating wheat; toxicity of different *Alternaria* compacta strains. Saudi J Biol Sci 26:210–215. 10.1016/j.sjbs.2018.10.007 30622428 10.1016/j.sjbs.2018.10.007 PMC6319088

[CR17] Ghosh S, Kruger C, Wicks S, Simon J, Kumar KG, Johnson WD, Mynatt RL, Noland RC, Richards BK (2016) Short chain acyl-CoA dehydrogenase deficiency and short-term high-fat diet perturb mitochondrial energy metabolism and transcriptional control of lipid-handling in liver. Nutr Metab (Lond) 13:17. 10.1186/s12986-016-0075-0 26933443 10.1186/s12986-016-0075-0 PMC4772307

[CR18] Hasuda AL, Person E, Khoshal AK, Bruel S, Puel S, Oswald IP, Bracarense APFRL, Pinton P (2022) Deoxynivalenol induces apoptosis and inflammation in the liver: analysis using precision-cut liver slices. Food Chem Toxicol 163:112930. 10.1016/j.fct.2022.112930 35314294 10.1016/j.fct.2022.112930

[CR19] Hellwig V, Grothe T, Mayer-Bartschmid A, Endermann R, Geschke F-U, Henkel T, Stadler M (2002) Altersetin, a new antibiotic from cultures of endophytic *Alternaria* spp. Taxonomy, fermentation, isolation, structure elucidation and biological activities. J Antibiot (Tokyo) 55:881–892. 10.7164/antibiotics.55.881 12523821 10.7164/antibiotics.55.881

[CR20] Hessel-Pras S, Kieshauer J, Roenn G, Luckert C, Braeuning A, Lampen A (2019) In vitro characterization of hepatic toxicity of *Alternaria* toxins. Mycotoxin Res 35:157–168. 10.1007/s12550-018-0339-9 30552586 10.1007/s12550-018-0339-9

[CR21] Hohenbichler J, Aichinger G, Rychlik M, Del Favero G, Marko D (2020) *Alternaria alternata* toxins synergistically activate the aryl hydrocarbon receptor pathway in vitro. Biomolecules 10:1018. 10.3390/biom10071018 32659980 10.3390/biom10071018 PMC7407958

[CR22] Jamnik T, Flasch M, Braun D, Fareed Y, Wasinger D, Seki D, Berry D, Berger A, Wisgrill L, Warth B (2022) Next-generation biomonitoring of the early-life chemical exposome in neonatal and infant development. Nat Commun 13:2653. 10.1038/s41467-022-30204-y 35550507 10.1038/s41467-022-30204-y PMC9098442

[CR23] Jiang M, Chen S, Li J, Liu L (2020) The biological and chemical diversity of tetramic acid compounds from marine-derived microorganisms. Mar Drugs 18:114. 10.3390/md18020114 32075282 10.3390/md18020114 PMC7074263

[CR24] Levy G, Jusko WJ (1966) Factors affecting the absorption of riboflavin in man. J Pharm Sci 55:285–289. 10.1002/jps.2600550305 5960174 10.1002/jps.2600550305

[CR25] Li S, Gao D, Jiang Y (2019) Function, detection and alteration of acylcarnitine metabolism in hepatocellular carcinoma. Metabolites. 10.3390/metabo9020036 30795537 10.3390/metabo9020036 PMC6410233

[CR26] Li Y, Shao Y, Zhu Y, Chen A, Qu J, Gao Y, Lu S, Luo P, Mao X (2023) Temperature-dependent mycotoxins production investigation in Alternaria infected cherry by ultra-high performance liquid chromatography and Orbitrap high resolution mass spectrometry. Int J Food Microbiol 388:110070. 10.1016/j.ijfoodmicro.2022.110070 36610234 10.1016/j.ijfoodmicro.2022.110070

[CR27] Louro H, Vettorazzi A, López de Cerain A, Spyropoulou A, Solhaug A, Straumfors A, Behr A-C, Mertens B, Žegura B, Fæste CK, Ndiaye D, Spilioti E, Varga E, Dubreil E, Borsos E, Crudo F, Eriksen GS, Snapkow I, Henri J, Sanders J, Machera K, Gaté L, Le Hegarat L, Novak M, Smith NM, Krapf S, Hager S, Fessard V, Kohl Y, Silva MJ, Dirven H, Dietrich J, Marko D (2024) Hazard characterization of *Alternaria* toxins to identify data gaps and improve risk assessment for human health. Arch Toxicol 98:425–469. 10.1007/s00204-023-03636-8 38147116 10.1007/s00204-023-03636-8 PMC10794282

[CR28] McCann MR, George De la Rosa MV, Rosania GR, Stringer KA (2021) l-carnitine and acylcarnitines: mitochondrial biomarkers for precision medicine. Metabolites 11:51. 10.3390/metabo11010051 33466750 10.3390/metabo11010051 PMC7829830

[CR29] Mosegaard S, Dipace G, Bross P, Carlsen J, Gregersen N, Olsen RKJ (2020) Riboflavin deficiency-implications for general human health and inborn errors of metabolism. Int J Mol Sci 21:3847. 10.3390/ijms21113847 32481712 10.3390/ijms21113847 PMC7312377

[CR30] Pang Z, Zhou G, Ewald J, Chang L, Hacariz O, Basu N, Xia J (2022) Using MetaboAnalyst 5.0 for LC–HRMS spectra processing, multi-omics integration and covariate adjustment of global metabolomics data. Nat Protoc 17:1735–1761. 10.1038/s41596-022-00710-w 35715522 10.1038/s41596-022-00710-w

[CR31] Pinton P, Oswald IP (2014) Effect of deoxynivalenol and other Type B trichothecenes on the intestine: a review. Toxins (Basel) 6:1615–1643. 10.3390/toxins6051615 24859243 10.3390/toxins6051615 PMC4052256

[CR32] Puntscher H, Kütt M-L, Skrinjar P, Mikula H, Podlech J, Fröhlich J, Marko D, Warth B (2018) Tracking emerging mycotoxins in food: development of an LC–MS/MS method for free and modified *Alternaria* toxins. Anal Bioanal Chem 410:4481–4494. 10.1007/s00216-018-1105-8 29766221 10.1007/s00216-018-1105-8 PMC6021461

[CR33] Puntscher H, Aichinger G, Grabher S, Attakpah E, Krüger F, Tillmann K, Motschnig T, Hohenbichler J, Braun D, Plasenzotti R, Pahlke G, Höger H, Marko D, Warth B (2019a) Bioavailability, metabolism, and excretion of a complex *Alternaria* culture extract versus altertoxin II: a comparative study in rats. Arch Toxicol 93:3153–3167. 10.1007/s00204-019-02575-7 31641809 10.1007/s00204-019-02575-7

[CR34] Puntscher H, Hankele S, Tillmann K, Attakpah E, Braun D, Kütt M-L, Del Favero G, Aichinger G, Pahlke G, Höger H, Marko D, Warth B (2019b) First insights into *Alternaria* multi-toxin in vivo metabolism. Toxicol Lett 301:168–178. 10.1016/j.toxlet.2018.10.006 30321595 10.1016/j.toxlet.2018.10.006

[CR35] Qin Q, Fan Y, Jia Q, Duan S, Liu F, Jia B, Wang G, Guo W, Wang C (2022) The potential of *Alternaria* toxins production by *A. alternata* in processing tomatoes. Toxins 14:827. 10.3390/toxins14120827 36548724 10.3390/toxins14120827 PMC9781988

[CR36] Schmutz C, Cenk E, Marko D (2019) The *Alternaria* mycotoxin alternariol triggers the immune response of IL-1β-stimulated, differentiated Caco-2 cells. Mol Nutr Food Res 63:1900341. 10.1002/mnfr.201900341 31584250 10.1002/mnfr.201900341 PMC6856692

[CR37] Schymanski EL, Jeon J, Gulde R, Fenner K, Ruff M, Singer HP, Hollender J (2014) Identifying small molecules via high resolution mass spectrometry: communicating confidence. Environ Sci Technol 48:2097–2098. 10.1021/es5002105 24476540 10.1021/es5002105

[CR38] Suwannasom N, Kao I, Pruß A, Georgieva R, Bäumler H (2020) Riboflavin: the health benefits of a forgotten natural vitamin. Int J Mol Sci 21:950. 10.3390/ijms21030950 32023913 10.3390/ijms21030950 PMC7037471

[CR39] Swigoňová Z, Mohsen A-W, Vockley J (2009) Acyl-CoA dehydrogenases: dynamic history of protein family evolution. J Mol Evol 69:176–193. 10.1007/s00239-009-9263-0 19639238 10.1007/s00239-009-9263-0 PMC4136416

[CR40] Tang N, Hong F, Hao W, Yu T-T, Wang G-G, Li W (2022) Riboflavin ameliorates mitochondrial dysfunction via the AMPK/PGC1α/HO-1 signaling pathway and attenuates carbon tetrachloride-induced liver fibrosis in rats. Exp Ther Med 24:608. 10.3892/etm.2022.11545 36160891 10.3892/etm.2022.11545 PMC9468838

[CR41] Thakur K, Tomar SK, Singh AK, Mandal S, Arora S (2017) Riboflavin and health: a review of recent human research. Crit Rev Food Sci Nutr 57:3650–3660. 10.1080/10408398.2016.1145104 27029320 10.1080/10408398.2016.1145104

[CR42] Tonazzi A, Mantovani C, Colella M, Terenghi G, Indiveri C (2013) Localization of mitochondrial carnitine/acylcarnitine translocase in sensory neurons from rat dorsal root ganglia. Neurochem Res 38:2535–2541. 10.1007/s11064-013-1168-z 24104610 10.1007/s11064-013-1168-z

[CR43] Tsugawa H, Cajka T, Kind T, Ma Y, Higgins B, Ikeda K, Kanazawa M, VanderGheynst J, Fiehn O, Arita M (2015) MS-DIAL: data-independent MS/MS deconvolution for comprehensive metabolome analysis. Nat Methods 12:523–526. 10.1038/nmeth.3393 25938372 10.1038/nmeth.3393 PMC4449330

[CR44] Udhayabanu T, Manole A, Rajeshwari M, Varalakshmi P, Houlden H, Ashokkumar B (2017) Riboflavin responsive mitochondrial dysfunction in neurodegenerative diseases. J Clin Med 6:52. 10.3390/jcm6050052 28475111 10.3390/jcm6050052 PMC5447943

[CR45] Watkins PA (2013) Fatty acyl-CoA synthetases. In: Lennarz WJ, Lane MD (eds) Encyclopedia of biological chemistry, 2nd edn. Academic Press, Waltham, pp 290–295. 10.1016/B978-0-12-378630-2.00100-6

[CR46] Wu F, Groopman JD, Pestka JJ (2014) Public health impacts of foodborne mycotoxins. Annu Rev Food Sci Technol 5:351–372. 10.1146/annurev-food-030713-092431 24422587 10.1146/annurev-food-030713-092431

[CR47] Xu G, Hansen JS, Zhao XJ, Chen S, Hoene M, Wang XL, Clemmesen JO, Secher NH, Häring HU, Pedersen BK, Lehmann R, Weigert C, Plomgaard P (2016) Liver and muscle contribute differently to the plasma acylcarnitine pool during fasting and exercise in humans. J Clin Endocrinol Metab 101:5044–5052. 10.1210/jc.2016-1859 27648961 10.1210/jc.2016-1859

[CR48] Yamada T, Tanaka A, Nehira T, Nishii T, Kikuchi T (2019) Altercrasins A−E, decalin derivatives, from a sea-urchin-derived *Alternaria* sp.: isolation and structural analysis including stereochemistry. Mar Drugs 17:218. 10.3390/md17040218 30978906 10.3390/md17040218 PMC6521173

[CR49] Yao Y, Yonezawa A, Yoshimatsu H, Omura T, Masuda S, Matsubara K (2013) Involvement of riboflavin transporter RFVT2/Slc52a2 in hepatic homeostasis of riboflavin in mice. Eur J Pharmacol 714:281–287. 10.1016/j.ejphar.2013.07.042 23911957 10.1016/j.ejphar.2013.07.042

[CR50] Yonezawa A, Inui K (2013) Novel riboflavin transporter family RFVT/SLC52: identification, nomenclature, functional characterization and genetic diseases of RFVT/SLC52. Mol Aspects Med ABCs Membr Transp Health Dis (SLC Ser) 34:693–701. 10.1016/j.mam.2012.07.014 10.1016/j.mam.2012.07.014 23506902

