# Supplementary material for: Rats exposed to Alternaria toxins in vivo exhibit altered liver activity highlighted by disruptions in riboflavin and acylcarnitine metabolism
Source: Arch Toxicol. 2024 Jun 28;98(10):3477–89. doi: 10.1007/s00204-024-03810-6 (PMC11402861; doi:10.1007/s00204-024-03810-6)
Supplement: Supplementary file 1 — Supplementary file1 (DOCX 3339 KB) [file 204_2024_3810_MOESM1_ESM.docx]

**SUPPLEMENTAL**

Rats Exposed to *Alternaria* Toxins *in vivo* Exhibit Altered Liver Activity Highlighted by Disruptions in Riboflavin and Acylcarnitine Metabolism

Jesse T. Peach^1^, Hannes Puntscher^1^, Harald Höger^2^, Doris Marko^1^ and Benedikt Warth^1,3,^*

1. Department of Food Chemistry and Toxicology, Faculty of Chemistry, University of Vienna, Vienna, Austria 2. Center for Biomedical Research, Medical University of Vienna, Vienna, Austria 3. Exposome Austria, Research Infrastructure and National EIRENE Node, Austria

***Corresponding Author**

Benedikt Warth: benedikt.warth@univie.ac.at

**Supplemental Table 1. Authentic reference standards examined for annotation.**

| **Name** | **CAS** | **Vendor** |
| --- | --- | --- |
| 2-Oxoglutaric acid | 328-50-7 | MetaSci |
| 3-Methylglutaric acid | 626-51-7 | MetaSci |
| 7-Dehydrocholesterol | 434-16-2 | MetaSci |
| Adenine | 2922-28-3 | MetaSci |
| Adenosine | 58-61-7 | MetaSci |
| Adenosine 5'-diphosphate (potassium salt) | 70285-70-0 | MetaSci |
| Adenosine 5'-monophosphate | 61-19-8 | MetaSci |
| Adenosine cyclophosphate | 60-92-4 | MetaSci |
| Adenosine triphosphate | 987-65-5 | MetaSci |
| Adipic acid | -648570 | MetaSci |
| Allantoin | 97-59-6 | MetaSci |
| alpha-Linolenic acid | 822-18-4 | MetaSci |
| Alternariol | 641-38-3 | TRC |
| Alternariol monomethyl ether | 26894-49-5 | TRC |
| Cholesterol | 57-88-5 | MetaSci |
| Citric acid | 77-92-9 | MetaSci |
| Creatine anhydrous | 57-00-1 | MetaSci |
| Creatine phosphate disodium salt tetrahydrate | 71519-72-7 | MetaSci |
| Creatinine | 60-27-5 | MetaSci |
| Cytosine | 71-30-7 | MetaSci |
| D-(+)-Glucose anhydrous | 50-99-7 | MetaSci |
| Dehydroascorbic acid | 490-83-5 | MetaSci |
| D-Fructose-1,6-bisphosphate (sodium salt hydrate) | 81028-91-3 | MetaSci |
| D-Fructose-6-phosphate disodium salt hydrate | 26177-86-6 | MetaSci |
| D-Glutamic acid | 6893-26-1 | MetaSci |
| DL-α-Lipoamide | 940-69-2 | MetaSci |
| D-Mannose | 3458-28-4 | MetaSci |
| D-Ribose | 50-69-1 | MetaSci |
| Flavin Adenine Dinucleotide disodium salt hydrate | 84366-81-4 | MetaSci |
| Fumaric acid | 110-17-8 | MetaSci |
| Glucose 1-phosphate | 150399-99-8 | MetaSci |
| Glucose 6-phosphate | 54010-71-8 | MetaSci |
| Glycerophosphocholine | 28319-77-9 | MetaSci |
| Glycine | 56-40-6 | MetaSci |
| Guanine | 73-40-5 | MetaSci |
| Guanosine | 118-00-3 | MetaSci |
| Guanosine 5′-triphosphate (sodium salt hydrate) | 56001-37-7 | MetaSci |
| Guanosine 5'-monophosphate (disodium salt) | 1333479 | MetaSci |
| Homocysteine | 454-29-5 | MetaSci |
| Hypoxanthine | 68-94-0 | MetaSci |
| Inosine | 58-63-9 | MetaSci |
| Inosine triphosphate | 35908-31-7 | MetaSci |
| Inosinic acid | 20813-76-7 | MetaSci |
| Isocitric acid | 1637-73-6 | MetaSci |
| Isopentanol | 123-51-3 | MetaSci |
| Isopentyl acetate | 123-92-2 | MetaSci |
| Kynurenic acid | 492-27-3 | MetaSci |
| L-Alanine | 56-41-7 | MetaSci |
| L-Arginine | 74-79-3 | MetaSci |
| L-Asparagine | 70-47-3 | MetaSci |
| L-Aspartic acid | 56-84-8 | MetaSci |
| L-Carnitine | 541-15-1 | MetaSci |
| L-Cysteine | 52-90-4 | MetaSci |
| L-Cystine | 56-89-3 | MetaSci |
| L-Glutamic acid | 56-86-0 | MetaSci |
| L-Glutamine | 56-85-9 | MetaSci |
| L-Glutathione oxidized | 27025-41-8 | MetaSci |
| L-Glutathione reduced | 70-18-8 | MetaSci |
| L-Histidine | 71-00-1 | MetaSci |
| L-Homoserine | 672-15-1 | MetaSci |
| Linoleic acid | 822-17-3 | MetaSci |
| L-Isoleucine | 73-32-5 | MetaSci |
| L-Kynurenine | 343-65-7 | MetaSci |
| L-Lactic acid | 79-33-4 | MetaSci |
| L-Leucine | 61-90-5 | MetaSci |
| L-Lysine | 56-87-1 | MetaSci |
| L-Methionine | 63-68-3 | MetaSci |
| L-Phenylalanine | 63-91-2 | MetaSci |
| L-Proline | 147-85-3 | MetaSci |
| L-Serine | 56-45-1 | MetaSci |
| L-Threonine | 72-19-5 | MetaSci |
| L-Tryptophan | 73-22-3 | MetaSci |
| L-Tyrosine | 60-18-4 | MetaSci |
| L-Valine | 72-18-4 | MetaSci |
| Maleic acid | 110-16-7 | MetaSci |
| Malic acid | 97-67-6 | MetaSci |
| Mannitol | 69-65-8 | MetaSci |
| Mevalonolactone | 674-26-0 | MetaSci |
| N-Acetylglucosamine | 7512-17-6 | MetaSci |
| NADH | 606-68-8 | MetaSci |
| NADP | 698999-85-8 | MetaSci |
| NADPH | 2646-71-1 | MetaSci |
| Oleic acid | 143-19-1 | MetaSci |
| Ornithine | 3184-13-2 | MetaSci |
| Oxalacetic acid | 328-42-7 | MetaSci |
| Oxalic acid | 144-62-7 | MetaSci |
| Palmitic acid | 57-10-3 | MetaSci |
| Pantothenic acid | 867-81-2 | MetaSci |
| Phospho(enol)pyruvic acid Monopotassium Salt | 4265-07-0 | MetaSci |
| Propionylcarnitine | 119793-66-7 | MetaSci |
| Pyroglutamic acid | 98-79-3 | MetaSci |
| Riboflavin | 83-88-5 | MetaSci |
| S-(5'-Adenosyl)-L-methionine *P-*toluenesulfonate salt | 52248-03-0 | MetaSci |
| Serotonin HCl | 153-98-0 | MetaSci |
| Sodium pyruvate | 113-24-6 | MetaSci |
| Spermidine | 124-20-9 | MetaSci |
| Squalene | 0111-02-04 | MetaSci |
| Succinic acid | 110-15-6 | MetaSci |
| Taurine | 107-35-7 | MetaSci |
| Thymine | 65-71-4 | MetaSci |
| Trimethylamine | 593-81-7 | MetaSci |
| Trimethylamine N-Oxide | 62637-93-8 | MetaSci |
| Uric acid | 69-93-2 | MetaSci |
| Vitamin C | 50-81-7 | MetaSci |
| Xanthine | 69-89-6 | MetaSci |
| Xanthosine dihydrate | 146-80-5 | MetaSci |
| β-nicotinamide adenine dinucleotide hydrate | 53-84-9 | MetaSci |
| β-Nicotinamide mononucleotide | 1094-61-7 | MetaSci |

**Supplemental Table 2. Annotations with identification level classification.**

| RT | m/z | Annotation | Identification Level | Reference Standard  Match | Labeled Yeast  Match | Spectral Library Score | *In-silico* Score | Mode | Adduct | CV | Signal/Noise | ANOVA *P-*value | FDR Corrected |
| --- | --- | --- | --- | --- | --- | --- | --- | --- | --- | --- | --- | --- | --- |
| 8.4 | 101.024 | Propionylformic acid | 2b |  |  | 81 |  | NEG | [M-H]- | 11 | 46 | 2.4E-01 | 9.1E-01 |
| 9.0 | 102.055 | 3-Aminoisobutyric acid | 2b |  |  | 84 |  | POS | [M+H]+ | 7 | 265 | 6.3E-01 | 9.3E-01 |
| 1.1 | 103.040 | Acetonic Acid | 2b |  |  | 84 |  | NEG | [M-H]- | 3 | 30 | 7.4E-01 | 9.3E-01 |
| 9.6 | 104.035 | Serine | 1 |  | Y |  | 99 | NEG | [M-H]- | 4 | 117 | 7.9E-01 | 9.4E-01 |
| 5.0 | 104.108 | Choline | 1 | Y |  | 78 |  | POS | [M+H]+ | 4 | 16308 | 5.1E-01 | 9.1E-01 |
| 3.8 | 105.020 | Glyceric acid | 3 |  |  |  | 99 | NEG | [M-H]- | 19 | 39 | 5.1E-01 | 9.1E-01 |
| 1.8 | 105.044 | 3-cyanopyridine | 2b |  |  | 86 |  | POS | [M+H]+ | 6 | 93 | 5.1E-02 | 6.6E-01 |
| 9.5 | 106.049 | Serine | 1 |  | Y | 100 |  | POS | [M+H]+ | 7 | 125 | 7.4E-01 | 9.3E-01 |
| 5.7 | 110.034 | Hypotaurine | 2b |  |  | 74 |  | POS | [M+H]+ | 4 | 1689 | 4.2E-01 | 9.1E-01 |
| 2.9 | 111.020 | 2,4-Dihydroxypyrimidine | 2B |  |  | 86 |  | NEG | [M-H]- | 4 | 557 | 4.4E-01 | 9.1E-01 |
| 2.5 | 113.035 | Uracil | 1 | Y |  |  |  | POS | [M+H]+ | 4 | 719 | 1.3E-01 | 8.6E-01 |
| 7.8 | 114.055 | L-Proline | 2b |  |  | 80 |  | NEG | [M-H]- | 3 | 65 | 7.5E-01 | 9.3E-01 |
| 6.6 | 114.066 | Creatinine | 1 | Y |  | 80 |  | POS | [M+H]+ | 13 | 50 | 3.5E-01 | 9.1E-01 |
| 12.0 | 115.088 | *N*-Nitrosopiperidine | 2b |  |  | 84 |  | POS | [M+H]+ | 19 | 183 | 8.0E-01 | 9.4E-01 |
| 7.8 | 116.070 | Proline | 1 | Y | Y | 86 |  | POS | [M+H]+ | 5 | 488 | 3.1E-01 | 9.1E-01 |
| 1.7 | 117.019 | Succinic acid | 2b |  |  | 84 |  | NEG | [M-H]- | 15 | 58 | 5.6E-01 | 9.2E-01 |
| 9.0 | 118.050 | alpha-Methyl-DL-serine | 2b |  |  | 85 |  | NEG | [M-H]- | 3 | 63 | 3.2E-01 | 9.1E-01 |
| 4.3 | 118.086 | 4-Methylmorpholine-N-oxide | 2b |  |  | 87 |  | POS | [M+H]+ | 14 | 43 | 4.2E-01 | 9.1E-01 |
| 7.0 | 118.086 | Valine | 1 | Y |  | 86 |  | POS | [M+H]+ | 15 | 20980 | 3.9E-01 | 9.1E-01 |
| 1.0 | 119.049 | Benzimidazole | 2b |  |  | 83 |  | POS | [M+H]+ | 9 | 74 | 3.9E-01 | 9.1E-01 |
| 7.5 | 119.072 | Methyl-3-hydroxybutyric acid | 2b |  |  | 78 |  | POS | [M+H]+ | 3 | 97 | 3.5E-01 | 9.1E-01 |
| 9.1 | 120.047 | 1*H*-Benzotriazole | 2b |  |  | 75 |  | POS | [M+H]+ | 10 | 173 | 4.4E-01 | 9.1E-01 |
| 9.0 | 120.065 | Homoserine | 2b |  |  | 71 |  | POS | [M+H]+ | 8 | 329 | 6.3E-01 | 9.3E-01 |
| 7.0 | 120.080 | Indoline | 2b |  |  | 84 |  | POS | [M+H]+ | 6 | 931 | 5.0E-01 | 9.1E-01 |
| 3.3 | 120.102 | Threonine | 2b |  |  | 78 |  | POS | [M+H]+ | 20 | 555 | 2.9E-01 | 9.1E-01 |
| 3.3 | 121.050 | Purine | 2b |  |  | 78 |  | POS | [M+H]+ | 8 | 119 | 3.3E-01 | 9.1E-01 |
| 1.7 | 122.096 | 2,4,6-Trimethylpyridine | 2b |  |  | 85 |  | POS | [M+H]+ | 10 | 790 | 5.8E-02 | 6.6E-01 |
| 1.9 | 122.096 | Phenylethylamine | 2b |  |  | 93 |  | POS | [M+H]+ | 3 | 855 | 1.6E-01 | 9.1E-01 |
| 1.5 | 122.110 | Hydrocarnitine | 3 |  |  |  | 67 | POS | [M+H]+ | 26 | 7 | 2.8E-01 | 9.1E-01 |
| 8.4 | 123.055 | Niacinamide | 2b |  |  | 85 |  | POS | [M+H]+ | 3 | 2619 | 9.4E-01 | 9.9E-01 |
| 4.2 | 123.092 | 4-(Dimethylamino)pyridine | 2b |  |  | 85 |  | POS | [M+H]+ | 5 | 503 | 9.1E-01 | 9.9E-01 |
| 7.5 | 124.010 | Taurine | 3 |  |  |  | 99 | NEG | [M-H]- | 7 | 474 | 4.6E-01 | 9.1E-01 |
| 1.4 | 124.039 | Nicotinic acid | 2b |  |  | 80 |  | POS | [M+H]+ | 18 | 254 | 4.9E-01 | 9.1E-01 |
| 8.2 | 126.021 | Taurine | 2b |  |  | 86 |  | POS | [M+H]+ | 11 | 107 | 4.9E-01 | 9.1E-01 |
| 10.2 | 127.038 | 3-Hydroxy-2-methyl-4-pyrone | 2b |  |  | 79 |  | POS | [M+H]+ | 17 | 28 | 1.0E+00 | 1.0E+00 |
| 9.6 | 127.051 | Thymine | 2b |  |  | 84 |  | NEG | [M-H]- | 4 | 74 | 5.5E-01 | 9.2E-01 |
| 3.7 | 128.035 | L-a-Aminoglutaric Acid Lactam | 2a |  |  | 87 | 92 | NEG | [M-H]- | 7 | 279 | 8.0E-01 | 9.4E-01 |
| 1.1 | 129.075 | Pyroglutamine | 3 |  |  |  | 57 | POS | [M+H]+ | 10 | 139 | 7.5E-01 | 9.3E-01 |
| 9.5 | 130.050 | delta-Aminolevulinic acid | 2b |  |  | 88 |  | POS | [M+H]+ | 7 | 193 | 7.0E-01 | 9.3E-01 |
| 6.9 | 130.086 | Leucine/Isoleucine | 1 | Y | Y | 80 |  | NEG | [M-H]- | 20 | 35 | 4.4E-01 | 9.1E-01 |
| 9.8 | 131.045 | Asparagine | 1 |  | Y |  |  | NEG | [M-H]- | 5 | 117 | 8.5E-01 | 9.7E-01 |
| 7.2 | 132.076 | Creatine | 2b |  |  | 79 |  | POS | [M+H]+ | 14 | 43 | 8.3E-01 | 9.6E-01 |
| 7.3 | 132.102 | Leucine/Isoleucine | 1 | Y | Y | 86 |  | POS | [M+H]+ | 13 | 571 | 9.7E-02 | 8.6E-01 |
| 13.8 | 133.059 | Gly-Gly | 2b |  |  | 71 |  | POS | [M+H]+ | 9 | 21 | 1.9E-01 | 9.1E-01 |
| 9.7 | 133.060 | Asparagine | 1 |  | Y | 100 |  | POS | [M+H]+ | 18 | 58 | 3.9E-01 | 9.1E-01 |
| 6.7 | 133.102 | Ornithine | 1 | Y |  | 97 |  | POS | [M+H]+ | 13 | 34 | 7.9E-01 | 9.4E-01 |
| 9.4 | 134.063 | 2-Oxindole | 2b |  |  | 73 |  | POS | [M+H]+ | 12 | 72 | 9.4E-01 | 9.9E-01 |
| 5.9 | 135.031 | Allopurinol | 2b |  |  | 91 |  | NEG | [M-H]- | 2 | 4586 | 3.6E-01 | 9.1E-01 |
| 2.8 | 136.039 | Homocysteine | 2b |  |  | 73 |  | POS | [M+H]+ | 8 | 950 | 4.7E-01 | 9.1E-01 |
| 5.7 | 136.061 | Adenine | 1 |  | Y | 74 |  | POS | [M+H]+ | 15 | 122 | 3.1E-01 | 9.1E-01 |
| 7.9 | 136.075 | 1,5,6,7-Tetrahydro-4H-indol-4-one | 2b |  |  | 84 |  | POS | [M+H]+ | 1 | 177 | 7.1E-01 | 9.3E-01 |
| 12.7 | 136.106 | 4-Propan-2-ylaniline | 2b |  |  | 76 |  | POS | [M+H]+ | 15 | 39 | 3.5E-01 | 9.1E-01 |
| 1.9 | 137.024 | 3-Hydroxybenzoic acid | 2b |  |  | 82 |  | NEG | [M-H]- | 19 | 292 | 5.5E-02 | 6.6E-01 |
| 5.7 | 137.046 | Hypoxanthine | 2b |  |  | 85 |  | POS | [M+H]+ | 5 | 61978 | 2.7E-01 | 9.1E-01 |
| 5.7 | 137.046 | Allopurinol | 2b |  |  | 82 |  | POS | [M+H]+ | 10 | 64187 | 6.3E-01 | 9.3E-01 |
| 6.2 | 137.071 | 4-Aminobenzamide | 2b |  |  | 85 |  | POS | [M+H]+ | 20 | 877 | 2.2E-01 | 9.1E-01 |
| 6.7 | 138.054 | Trigonelline | 2b |  |  | 70 |  | POS | [M+H]+ | 3 | 332 | 4.9E-01 | 9.1E-01 |
| 7.3 | 138.054 | Methyl nicotinic acid | 2b |  |  | 71 |  | POS | [M+H]+ | 13 | 248 | 4.1E-01 | 9.1E-01 |
| 7.6 | 138.055 | Homarine | 2b |  |  | 71 |  | POS | [M+H]+ | 10 | 460 | 1.2E-01 | 8.6E-01 |
| 2.9 | 138.091 | *P-*phenetidine | 2b |  |  | 71 |  | POS | [M+H]+ | 9 | 84 | 2.5E-01 | 9.1E-01 |
| 11.0 | 141.078 | 2-Methyl-4-amino-6-methoxy-s-triazine | 2b |  |  | 85 |  | POS | [M+H]+ | 8 | 37 | 4.4E-02 | 6.6E-01 |
| 12.7 | 144.047 | 4-Hydroxyquinoline | 2b |  |  | 83 |  | POS | [M+H]+ | 16 | 2847 | 1.3E-01 | 8.6E-01 |
| 6.9 | 144.101 | Homoproline methyl ester | 2b |  |  | 83 |  | POS | [M+H]+ | 2 | 538 | 2.1E-01 | 9.1E-01 |
| 9.6 | 145.061 | Glutamine | 1 |  | Y | 94 |  | NEG | [M-H]- | 5 | 126 | 3.3E-01 | 9.1E-01 |
| 7.1 | 146.060 | 3-Formylindole | 2b |  |  | 84 |  | POS | [M+H]+ | 2 | 109 | 7.3E-01 | 9.3E-01 |
| 5.2 | 146.117 | Acetylcholine | 2b |  |  | 99 |  | POS | [M+H]+ | 5 | 106 | 6.1E-01 | 9.3E-01 |
| 9.5 | 147.076 | Glutamine | 1 |  | Y | 81 |  | POS | [M+H]+ | 6 | 119 | 6.8E-01 | 9.3E-01 |
| 1.5 | 147.092 | Dimethylbenzimidazole | 2b |  |  | 78 |  | POS | [M+H]+ | 9 | 59 | 5.5E-01 | 9.2E-01 |
| 5.1 | 149.045 | Ribose | 1 | Y |  |  |  | NEG | [M-H]- | 3 | 27 | 6.6E-01 | 9.3E-01 |
| 7.5 | 150.058 | Methionine | 2b |  |  | 88 | 99 | POS | [M+H]+ | 14 | 20 | 9.8E-02 | 8.6E-01 |
| 6.4 | 151.026 | Xanthine | 2b |  |  | 90 |  | NEG | [M-H]- | 3 | 4003 | 3.9E-01 | 9.1E-01 |
| 6.7 | 151.030 | 2-Mercaptobenzimidazole | 2b |  |  | 78 |  | POS | [M+H]+ | 2 | 75 | 2.7E-01 | 9.1E-01 |
| 7.7 | 152.056 | Guanine | 1 | Y | Y | 100 |  | POS | [M+H]+ | 9 | 4021 | 4.3E-01 | 9.1E-01 |
| 8.9 | 152.073 | 2-Phenylglycine | 2b |  |  | 84 |  | POS | [M+H]+ | 15 | 94 | 3.9E-01 | 9.1E-01 |
| 6.2 | 153.041 | Xanthine | 1 | Y |  | 91 |  | POS | [M+H]+ | 4 | 1941 | 9.9E-01 | 1.0E+00 |
| 2.7 | 153.065 | N1-Methyl-4-pyridone-3-carboxamide | 2b |  |  | 83 |  | POS | [M+H]+ | 4 | 482 | 4.9E-01 | 9.1E-01 |
| 5.9 | 153.101 | Pyrimidinol | 2b |  |  | 84 |  | POS | [M+H]+ | 8 | 15 | 1.1E-01 | 8.6E-01 |
| 5.8 | 154.077 | Dopamine | 2b |  |  | 72 |  | POS | [M+H]+ | 16 | 226 | 5.3E-01 | 9.1E-01 |
| 5.0 | 154.122 | Gabapentin-lactam | 2b |  |  | 75 |  | POS | [M+H]+ | 1 | 82 | 6.6E-01 | 9.3E-01 |
| 13.6 | 156.076 | Histidine | 1 | Y | Y | 87 |  | POS | [M+H]+ | 9 | 92 | 5.3E-01 | 9.1E-01 |
| 11.0 | 157.073 | 4,4'-Bipyridine | 2b |  |  | 78 |  | POS | [M+H]+ | 4 | 36 | 7.4E-01 | 9.3E-01 |
| 7.8 | 160.034 | Trigonelline | 2b |  |  | 84 |  | POS | [M+Na]+ | 18 | 121 | 9.0E-01 | 9.8E-01 |
| 1.2 | 160.133 | Pregabalin | 2a |  |  | 82 | 83 | POS | [M+H]+ | 6 | 80 | 1.9E-01 | 9.1E-01 |
| 10.3 | 161.045 | Hydroxymethylglutaric acid | 2b |  |  | 84 |  | NEG | [M-H]- | 1 | 521 | 9.9E-01 | 1.0E+00 |
| 1.6 | 162.054 | 2,8-Quinolinediol | 2b |  |  | 74 |  | POS | [M+H]+ | 9 | 57 | 3.8E-01 | 9.1E-01 |
| 6.6 | 162.112 | Carnitine | 1 | Y |  | 81 | 93 | POS | [M+H]+ | 10 | 224 | 9.5E-01 | 9.9E-01 |
| 7.9 | 163.039 | *P-*Coumaric acid | 2b |  |  | 91 |  | NEG | [M-H]- | 7 | 29 | 3.6E-01 | 9.1E-01 |
| 9.0 | 164.040 | Methionine sulfoxide | 3 |  |  |  | 99 | NEG | [M-H]- | 22 | 42 | 7.3E-01 | 9.3E-01 |
| 1.8 | 164.052 | 1H-Benzotriazole-5-carboxylic acid | 2b |  |  | 70 |  | POS | [M+H]+ | 19 | 222 | 2.7E-01 | 9.1E-01 |
| 7.9 | 165.054 | 2-Norbornene-5,6-dicarboxylic anhydride | 2b |  |  | 72 |  | POS | [M+H]+ | 1 | 191 | 6.7E-01 | 9.3E-01 |
| 9.8 | 165.097 | Fenuron | 2b |  |  | 76 |  | POS | [M+H]+ | 6 | 503 | 8.9E-01 | 9.8E-01 |
| 7.0 | 166.086 | Phenylalanine | 1 | Y |  | 89 | 99 | POS | [M+H]+ | 7 | 586 | 2.4E-01 | 9.1E-01 |
| 5.8 | 167.042 | Thiopurine S-methylether | 2b |  |  | 73 |  | POS | [M+H]+ | 12 | 129 | 3.4E-01 | 9.1E-01 |
| 5.5 | 168.070 | Pyridoxine | 3 |  |  |  | 85 | NEG | [M-H]- | 19 | 14 | 7.2E-01 | 9.3E-01 |
| 1.3 | 170.120 | Hydroindol | 3 |  |  |  | 57 | POS | [M+H]+ | 6 | 364 | 6.2E-01 | 9.3E-01 |
| 4.0 | 172.132 | Gabapentin | 2b |  |  | 72 |  | POS | [M+H]+ | 10 | 31 | 2.4E-01 | 9.1E-01 |
| 4.8 | 174.050 | Atrazine-desisopropyl | 2b |  |  | 70 |  | POS | [M+H]+ | 8 | 121 | 9.5E-01 | 9.9E-01 |
| 7.2 | 175.120 | Arginine | 1 | Y |  |  |  | POS | [M+H]+ | 12 | 16 | 9.1E-01 | 9.9E-01 |
| 5.7 | 177.046 | Fluxapyroxad | 2b |  |  | 82 |  | POS | [M+H]+ | 12 | 44 | 3.3E-01 | 9.1E-01 |
| 7.9 | 179.055 | Glucose | 1 | Y |  |  |  | NEG | [M-H]- | 9 | 22 | 7.1E-01 | 9.3E-01 |
| 5.3 | 179.062 | 4-(Dimethylamino)phenylthiocyanate | 2b |  |  | 70 |  | POS | [M+H]+ | 3 | 2443 | 3.9E-01 | 9.1E-01 |
| 7.9 | 180.066 | Tyrosine | 1 |  | Y | 90 |  | NEG | [M-H]- | 3 | 279 | 5.0E-01 | 9.1E-01 |
| 12.5 | 180.135 | N-Methylephedrine | 2b |  |  | 77 |  | POS | [M+H]+ | 13 | 185 | 4.1E-01 | 9.1E-01 |
| 0.9 | 181.079 | 1,10-Phenanthroline | 2b |  |  | 72 |  | POS | [M+H]+ | 7 | 558 | 4.1E-01 | 9.1E-01 |
| 4.9 | 182.045 | 4-Pyridoxate | 2b |  |  | 85 |  | NEG | [M-H]- | 18 | 279 | 2.7E-01 | 9.1E-01 |
| 7.9 | 182.081 | Tyrosine | 1 | Y |  | 92 |  | POS | [M+H]+ | 11 | 230 | 6.8E-01 | 9.3E-01 |
| 5.8 | 183.065 | Homovanillic acid | 2b |  |  | 81 |  | POS | [M+H]+ | 12 | 14 | 5.0E-01 | 9.1E-01 |
| 6.4 | 184.070 | Phosphocholine | 3 |  |  |  | 78 | POS | [M+H]+ | 6 | 65 | 3.9E-01 | 9.1E-01 |
| 9.2 | 187.078 | 3,3`-Biphenol | 2b |  |  | 74 |  | POS | [M+H]+ | 16 | 67 | 3.0E-01 | 9.1E-01 |
| 1.5 | 188.066 | Atrazine-desethyl | 2b |  |  | 78 |  | POS | [M+H]+ | 11 | 131 | 8.5E-01 | 9.7E-01 |
| 7.1 | 188.070 | Deethylatrazine | 2b |  |  | 87 |  | POS | [M+H]+ | 3 | 213 | 7.5E-01 | 9.3E-01 |
| 12.2 | 188.073 | 3-Indoleacrylic acid | 2b |  |  | 79 |  | POS | [M+H]+ | 22 | 15 | 1.1E-01 | 8.6E-01 |
| 6.1 | 189.123 | Ala-Val | 2b |  |  | 70 |  | POS | [M+H]+ | 25 | 13 | 2.7E-01 | 9.1E-01 |
| 14.5 | 194.118 | Isoprocarb | 2b |  |  | 79 |  | POS | [M+H]+ | 11 | 44 | 6.9E-01 | 9.3E-01 |
| 0.7 | 197.096 | 1,2-Diphenylethanone | 2b |  |  | 76 |  | POS | [M+H]+ | 13 | 48 | 3.5E-01 | 9.1E-01 |
| 0.7 | 201.163 | Isolongifolene, 4,5,9,10-dehydro- | 3 |  |  |  | 60 | POS | [M+H]+ | 4 | 35 | 4.6E-01 | 9.1E-01 |
| 5.9 | 202.085 | Simazine | 2b |  |  | 70 |  | POS | [M+H]+ | 6 | 19 | 3.9E-01 | 9.1E-01 |
| 6.2 | 202.180 | 4-Hydroxy-1-(2-hydroxyethyl)-2,2,6,6-tetramethylpiperidine | 2b |  |  | 80 |  | POS | [M+H]+ | 6 | 1301 | 5.0E-01 | 9.1E-01 |
| 8.2 | 203.053 | Diphenylphosphine oxide | 2b |  |  | 73 |  | POS | [M+H]+ | 11 | 1376 | 8.0E-01 | 9.4E-01 |
| 6.4 | 203.080 | Tryptophan | 3 |  |  |  | 99 | NEG | [M-H]- | 26 | 17 | 8.8E-01 | 9.7E-01 |
| 7.4 | 204.086 | N-Acetyl-D-galactosamine | 2b |  |  | 77 |  | POS | [M+H-H2O]+ | 13 | 123 | 9.5E-01 | 9.9E-01 |
| 3.4 | 204.105 | 7-(Dimethylamino)-4-methylcoumarin | 2b |  |  | 77 |  | POS | [M+H]+ | 6 | 169 | 8.3E-01 | 9.6E-01 |
| 2.7 | 204.122 | Isopentenyladenine | 2b |  |  | 74 |  | POS | [M+H]+ | 0 | 502 | 1.1E-01 | 8.6E-01 |
| 8.0 | 205.068 | Sorbitol | 2b |  |  | 77 |  | POS | [M+Na]+ | 8 | 407 | 5.2E-01 | 9.1E-01 |
| 5.9 | 206.048 | 2-Acrylamido-2-methyl-1-propanesulfonic acid | 2b |  |  | 83 |  | NEG | [M-H]- | 12 | 15 | 8.0E-01 | 9.4E-01 |
| 4.8 | 207.012 | 2-Naphthalenesulfonic acid | 2b |  |  | 86 |  | NEG | [M-H]- | 10 | 230 | 9.8E-01 | 1.0E+00 |
| 5.8 | 207.057 | (4S,5Z,6S)-4-(2-methoxy-2-oxoethyl)-5-[2-[(E)-3-phenylpro*P-*2-enoyl]oxyethylidene]-6-[(2S,3R,4S,5S,6R)-3,4,5-trihydroxy-6-(hydroxymethyl)oxan-2-yl]oxy-4H-pyran-3-carboxylic acid | 2b |  |  | 72 |  | POS | [M+H]+ | 8 | 22414 | 4.6E-01 | 9.1E-01 |
| 0.8 | 209.150 | 4-5-Dihydrovomifoliol | 3 |  |  |  | 70 | POS | [M+H]+ | 2 | 37 | 4.8E-01 | 9.1E-01 |
| 8.9 | 211.082 | Pyocyanin | 2b |  |  | 82 |  | NEG | [M-H]- | 6 | 486 | 9.2E-01 | 9.9E-01 |
| 2.0 | 212.118 | 1,3-Diphenylguanidine | 2b |  |  | 82 |  | POS | [M+H]+ | 16 | 491 | 8.6E-01 | 9.7E-01 |
| 1.9 | 216.981 | 5-Sulfosalicylic acid | 3 |  |  |  | 48 | NEG | [M-H]- | 19 | 1172 | 3.6E-02 | 6.0E-01 |
| 3.2 | 218.100 | Pantothenic acid | 3 |  |  |  | 99 | NEG | [M-H]- | 7 | 1946 | 1.3E-01 | 8.6E-01 |
| 2.5 | 218.138 | Propionylcarnitine | 1 | Y |  |  | 38 | POS | [M+H]+ | 14 | 98 | 3.2E-03 | 5.4E-02 |
| 1.6 | 220.064 | N-lactoyl-Methionine | 3 |  |  |  | 80 | NEG | [M-H]- | 12 | 59 | 7.2E-02 | 7.9E-01 |
| 2.6 | 220.117 | trans-Zeatin | 2b |  |  | 73 |  | POS | [M+H]+ | 14 | 772 | 4.6E-01 | 9.1E-01 |
| 1.3 | 224.073 | 2-Aminoanthraquinone | 2b |  |  | 71 |  | POS | [M+H]+ | 6 | 347 | 5.5E-01 | 9.2E-01 |
| 4.5 | 224.080 | Norketamine | 2b |  |  | 79 | 99 | POS | [M+H]+ | 1 | 6953 | 2.7E-01 | 9.1E-01 |
| 0.7 | 225.091 | Etofylline | 2b |  |  | 77 |  | POS | [M+H]+ | 20 | 106 | 4.8E-01 | 9.1E-01 |
| 12.0 | 230.098 | Benz[c]acridine | 2b |  |  | 77 |  | POS | [M+H]+ | 6 | 186 | 8.8E-01 | 9.7E-01 |
| 6.1 | 231.152 | Propyphenazone | 2b |  |  | 72 |  | POS | [M+H]+ | 30 | 264 | 9.3E-01 | 9.9E-01 |
| 3.4 | 232.136 | 7-Diethylamino-4-methylcoumarin | 2b |  |  | 72 |  | POS | [M+H]+ | 10 | 111 | 5.8E-01 | 9.3E-01 |
| 2.0 | 232.154 | Butyrylcarnitine | 3 |  |  |  | 47 | POS | [M+H]+ | 11 | 112 | 8.6E-04 | 5.3E-02 |
| 8.8 | 235.079 | Perseitol | 2b |  |  | 78 |  | POS | [M+Na]+ | 13 | 219 | 6.3E-01 | 9.3E-01 |
| 0.9 | 235.090 | (2R)-5-methoxy-2-methyl-2,3,8,9-tetrahydrofuro[2,3-h]chromen-4-one | 2b |  |  | 73 |  | POS | [M+H]+ | 11 | 1294 | 6.9E-01 | 9.3E-01 |
| 2.6 | 237.105 | Carbamazepine | 2b |  |  | 76 |  | POS | [M+H]+ | 1 | 1743 | 2.9E-01 | 9.1E-01 |
| 2.4 | 238.100 | Ketamine | 2a |  |  | 86 | 99 | POS | [M+H]+ | 15 | 11891 | 1.2E-01 | 8.6E-01 |
| 1.4 | 239.148 | Pentaethylene glycol | 2a |  |  | 82 | 99 | POS | [M+H]+ | 10 | 273 | 3.4E-01 | 9.1E-01 |
| 6.7 | 240.078 | Clomazone | 2b |  |  | 73 |  | POS | [M+H]+ | 6 | 580 | 3.8E-01 | 9.1E-01 |
| 5.8 | 243.062 | Uridine | 2b |  |  | 92 |  | NEG | [M-H]- | 14 | 5048 | 6.2E-01 | 9.3E-01 |
| 1.6 | 243.087 | Lumichrome | 2b |  |  | 74 |  | POS | [M+H]+ | 10 | 239 | 5.9E-01 | 9.3E-01 |
| 7.5 | 244.078 | *N*-acetyl-D-mannosamine | 2a |  |  | 76 | 99 | POS | [M+Na]+ | 3 | 889 | 5.7E-01 | 9.3E-01 |
| 8.8 | 244.093 | Cytidine | 2b |  |  | 85 |  | POS | [M+H]+ | 7 | 171 | 2.8E-02 | 5.5E-01 |
| 1.1 | 251.085 | 13-methoxy-1,6-diazatetracyclohexadeca-3,5,7,9(16),10,12,14-heptaen-2-one | 2b |  |  | 71 |  | POS | [M+H]+ | 4 | 326 | 3.8E-01 | 9.1E-01 |
| 1.7 | 251.120 | Methaquaione | 2b |  |  | 73 |  | POS | [M+H]+ | 23 | 658 | 7.4E-01 | 9.3E-01 |
| 3.0 | 253.100 | (2S)-2-[(1S,2R)-1,2-Dihydroxypentyl]-4-methoxy-2,3-dihydropyran-6-one | 2b |  |  | 74 |  | POS | [M+Na]+ | 6 | 102 | 3.4E-01 | 9.1E-01 |
| 8.4 | 255.097 | Dihydrokavain | 2b |  |  | 73 |  | POS | [M+Na]+ | 6 | 456 | 9.5E-01 | 9.9E-01 |
| 5.1 | 256.114 | Dimethachlor | 2b |  |  | 83 |  | POS | [M+H]+ | 12 | 9 | 2.8E-01 | 9.1E-01 |
| 10.1 | 258.110 | 5-Methylcytidine | 1 | Y |  | 80 |  | POS | [M+H]+ | 7 | 493 | 5.7E-02 | 6.6E-01 |
| 1.4 | 261.130 | Diaveridine | 2b |  |  | 77 |  | POS | [M+H]+ | 7 | 256 | 3.6E-01 | 9.1E-01 |
| 8.7 | 263.074 | 2-Phenylfuro[2,3-h]chromen-4-one | 2b |  |  | 77 |  | POS | [M+H]+ | 11 | 50 | 6.5E-01 | 9.3E-01 |
| 12.7 | 265.111 | Thiamine | 2b |  |  | 81 |  | POS | [M]+ | 22 | 38 | 1.9E-01 | 9.1E-01 |
| 6.0 | 267.072 | Inosine | 1 | Y |  | 82 |  | NEG | [M-H]- | 3 | 63 | 9.1E-02 | 8.6E-01 |
| 6.9 | 268.103 | Adenosine | 1 | Y |  | 83 |  | POS | [M+H]+ | 9 | 303 | 9.4E-01 | 9.9E-01 |
| 6.6 | 269.087 | Inosine | 2b |  |  | 81 |  | POS | [M+H]+ | 4 | 390 | 7.8E-01 | 9.4E-01 |
| 1.1 | 269.094 | 3-Hydroxybenzo(a)pyrene | 2b |  |  | 72 |  | POS | [M+H]+ | 7 | 23 | 1.9E-01 | 9.1E-01 |
| 1.5 | 273.169 | 3-Hydroxytetradecanedioic acid | 3 |  |  |  | 65 | NEG | [M-H]- | 12 | 60 | 3.3E-01 | 9.1E-01 |
| 1.5 | 274.273 | Lauryldiethanolamine | 2b |  |  | 83 |  | POS | [M+H]+ | 19 | 217 | 9.0E-01 | 9.8E-01 |
| 7.8 | 277.115 | Ursinoic Acid | 2b |  |  | 71 |  | POS | [M+H]+ | 10 | 28 | 2.4E-01 | 9.1E-01 |
| 4.9 | 279.093 | Sulfamethazine | 2b |  |  | 71 |  | POS | [M+H]+ | 11 | 24 | 4.3E-01 | 9.1E-01 |
| 7.9 | 282.083 | Guanosine | 1 | Y |  |  |  | NEG | [M-H]- | 4 | 26 | 4.7E-01 | 9.1E-01 |
| 9.2 | 282.154 | Coumarin | 2b |  |  | 79 |  | POS | [M+H]+ | 12 | 72 | 2.2E-01 | 9.1E-01 |
| 0.8 | 282.279 | Oleamide | 2b |  |  | 81 |  | POS | [M+H]+ | 9 | 1179 | 5.3E-01 | 9.1E-01 |
| 6.8 | 283.067 | Wogonin | 2b |  |  | 81 |  | NEG | [M-H]- | 4 | 684 | 1.8E-01 | 9.1E-01 |
| 7.1 | 283.067 | Acacetin | 2b |  |  | 80 |  | NEG | [M-H]- | 5 | 732 | 6.2E-01 | 9.3E-01 |
| 4.5 | 291.199 | (2'E,4'Z,7'Z,8E)-Colnelenic acid | 3 |  |  |  | 35 | NEG | [M-H]- | 9 | 210 | 6.8E-01 | 9.3E-01 |
| 1.1 | 293.178 | Tetradecyl sulfate | 2b |  |  | 81 |  | NEG | [M-H]- | 8 | 266 | 8.6E-01 | 9.7E-01 |
| 5.7 | 298.098 | 5'-S-Methylthioadenosine | 1 | Y |  | 99 | 82 | POS | [M+H]+ | 6 | 212 | 6.5E-01 | 9.3E-01 |
| 1.0 | 299.199 | 3,4-Didehydroretinoic acid | 3 |  |  |  | 70 | POS | [M+H]+ | 10 | 45 | 1.7E-01 | 9.1E-01 |
| 7.6 | 300.198 | Nylidrin | 2b |  |  | 72 |  | POS | [M+H]+ | 7 | 43 | 7.5E-01 | 9.3E-01 |
| 5.1 | 300.290 | Sphingosine | 3 |  |  |  | 93 | POS | [M+H]+ | 2 | 37 | 3.6E-01 | 9.1E-01 |
| 4.2 | 315.047 | Isorhamnetin | 2b |  |  | 83 |  | NEG | [M-H]- | 7 | 435 | 4.4E-01 | 9.1E-01 |
| 3.3 | 317.062 | Isorhamnetin | 2b |  |  | 75 |  | POS | [M+H]+ | 7 | 138 | 5.3E-01 | 9.1E-01 |
| 5.1 | 322.270 | Palmitoylethenolamide | 3 |  |  |  | 75 | POS | [M+H]+ | 11 | 15 | 3.4E-01 | 9.1E-01 |
| 10.2 | 325.112 | Prazepam | 2b |  |  | 73 |  | POS | [M+H]+ | 9 | 176 | 8.9E-01 | 9.7E-01 |
| 1.7 | 325.183 | Dodecylbenzenesulfonic acid | 2b |  |  | 84 |  | NEG | [M-H]- | 24 | 732 | 5.5E-01 | 9.2E-01 |
| 1.5 | 327.201 | Ajmaline | 2b |  |  | 76 |  | POS | [M+H]+ | 9 | 1193 | 6.3E-01 | 9.3E-01 |
| 0.8 | 327.232 | Docosahexaenoic acid | 2b |  |  | 83 |  | NEG | [M-H]- | 26 | 549 | 3.4E-01 | 9.1E-01 |
| 4.5 | 337.272 | Isolinderanolide | 3 |  |  |  | 57 | POS | [M+H]+ | 13 | 68 | 1.1E-01 | 8.6E-01 |
| 10.3 | 341.107 | Trehalose | 2b |  |  | 87 |  | NEG | [M-H]- | 1 | 760 | 7.5E-01 | 9.3E-01 |
| 6.0 | 341.240 | Methyl arachidonate | 3 |  |  |  | 64 | POS | [M+H]+ | 24 | 27 | 1.0E+00 | 1.0E+00 |
| 8.4 | 346.160 | (E)-1,7-bis(3,4-dihydroxyphenyl)hept-4-en-3-one | 2b |  |  | 76 |  | POS | [M+NH4]+ | 15 | 6 | 2.1E-01 | 9.1E-01 |
| 1.0 | 346.280 | Retinol acetate | 3 |  |  |  | 61 | POS | [M+H]+ | 1 | 21 | 4.6E-01 | 9.1E-01 |
| 1.0 | 358.238 | 11-(2H-1,3-benzodioxol-5-yl)-N-(2-methylpropyl)undec-10-enamide | 3 |  |  |  | 84 | NEG | [M-H]- | 15 | 30 | 3.8E-01 | 9.1E-01 |
| 10.3 | 360.149 | Turanose | 2b |  |  | 72 |  | POS | [M+NH4]+ | 11 | 96 | 7.9E-01 | 9.4E-01 |
| 9.8 | 365.105 | Sucrose | 2b |  |  | 80 |  | POS | [M+Na]+ | 9 | 27 | 4.3E-01 | 9.1E-01 |
| 10.3 | 365.106 | Melibiose | 2b |  |  | 88 |  | POS | [M+Na]+ | 5 | 126 | 8.6E-01 | 9.7E-01 |
| 8.4 | 365.107 | Trehalose | 2b |  |  | 75 |  | POS | [M+Na]+ | 15 | 76 | 8.8E-01 | 9.7E-01 |
| 0.9 | 366.210 | Arazine | 3 |  |  |  | 79 | NEG | [M-H]- | 29 | 15 | 6.6E-01 | 9.3E-01 |
| 1.5 | 371.227 | Aspidocarpine | 2b |  |  | 71 |  | POS | [M+H]+ | 7 | 994 | 7.1E-01 | 9.3E-01 |
| 6.9 | 375.129 | Riboflavin | 1 | Y |  | 100 |  | NEG | [M-H]- | 1 | 70 | 3.5E-04 | 4.8E-02 |
| 1.1 | 377.190 | 4-5-Dihydronivensin | 3 |  |  |  | 66 | POS | [M+H]+ | 14 | 16 | 9.8E-01 | 1.0E+00 |
| 4.3 | 386.136 | Sarafloxacin | 2b |  |  | 76 |  | POS | [M+H]+ | 8 | 6057 | 8.4E-01 | 9.6E-01 |
| 4.4 | 387.108 | Methyl 2-(9,10a-dihydroxy-4a-methoxy-1-methyl-5,10-dioxo-3,4-dihydro-1H-benzo[g]isochromen-3-yl)acetate | 2b |  |  | 76 |  | POS | [M+Na]+ | 13 | 4 | 2.8E-01 | 9.1E-01 |
| 0.7 | 390.230 | Nileprost | 3 |  |  |  | 55 | NEG | [M-H]- | 26 | 24 | 3.4E-01 | 9.1E-01 |
| 6.1 | 391.141 | (2S)-8-[(E)-3-Hydroxy-3-methylbut-1-enyl]-5,7-dimethoxy-2-phenyl-2,3-dihydrochromen-4-one | 2b |  |  | 72 |  | POS | [M+H]+ | 20 | 6 | 6.7E-01 | 9.3E-01 |
| 1.5 | 393.209 | Octaethylene glycol | 2b |  |  | 74 |  | POS | [M+Na]+ | 5 | 629 | 8.1E-01 | 9.4E-01 |
| 0.8 | 398.233 | Altersetin | 2a |  |  | 86 | 61 | NEG | [M-H]- | 20 | 187 | 8.7E-05 | 7.6E-03 |
| 1.0 | 399.146 | S-(5'-Adenosyl)-L-methionine | 1 | Y |  |  |  | POS | [M+H]+ | 2 | 26 | 6.3E-01 | 9.3E-01 |
| 1.0 | 400.247 | Altersetin | 3 |  |  |  | 62 | POS | [M+H]+ | 3 | 50 | 1.2E-06 | 1.6E-04 |
| 2.9 | 410.230 | LysoPC(10:0/0:0) | 3 |  |  |  | 80 | NEG | [M-H]- | 21 | 28 | 1.0E-01 | 8.6E-01 |
| 7.0 | 411.120 | Rhinoclactone D | 3 |  |  |  | 78 | NEG | [M-H]- | 4 | 109 | 5.0E-03 | 1.9E-01 |
| 5.2 | 414.095 | 2-(Glutathione-S-yl)-hydroquinone | 3 |  |  |  | 63 | NEG | [M-H]- | 10 | 370 | 1.9E-02 | 4.5E-01 |
| 1.0 | 414.226 | Altercrasin A | 3 |  |  |  | 97 | NEG | [M-H]- | 12 | 142 | 1.2E-11 | 3.2E-09 |
| 5.0 | 414.270 | Bimatoprost | 3 |  |  |  | 33 | NEG | [M-H]- | 12 | 15 | 7.5E-01 | 9.3E-01 |
| 4.0 | 426.321 | Cholic Acid | 2b |  |  | 74 |  | POS | [M+NH4]+ | 9 | 38 | 2.7E-01 | 9.1E-01 |
| 9.3 | 431.141 | Formononetin-7-O-glucoside | 2b |  |  | 75 |  | POS | [M+H]+ | 12 | 89 | 2.8E-01 | 9.1E-01 |
| 4.5 | 432.310 | Glycochenodeoxycholic acid | 2b |  |  | 75 |  | POS | [M+H-H2O]+ | 7 | 80 | 1.3E-01 | 8.6E-01 |
| 7.4 | 433.150 | Tryptoquivaline Q | 3 |  |  |  | 67 | NEG | [M-H]- | 23 | 14 | 7.3E-01 | 9.3E-01 |
| 5.0 | 434.240 | Paxilline | 3 |  |  |  | 57 | NEG | [M-H]- | 10 | 71 | 7.1E-01 | 9.3E-01 |
| 4.6 | 444.240 | 4,6',7'-Trihydroxy-7-(2-hydroxyethyl)-2',5',5',8'a-tetramethyl-3,3',4',4'a,5',6,6',7,7',8,8',8'a-dodecahydro-2'H-spiro[furo[2,3-e]isoindole-2,1'-naphthalen]-6-one | 3 |  |  |  | 64 | NEG | [M-H]- | 10 | 12 | 6.4E-01 | 9.3E-01 |
| 5.5 | 447.132 | Glycitin | 2b |  |  | 82 |  | NEG | [M-H]- | 17 | 1303 | 4.5E-01 | 9.1E-01 |
| 3.0 | 448.305 | Chenodeoxyglycocholic acid | 2b |  |  | 90 |  | NEG | [M-H]- | 8 | 87 | 8.3E-02 | 8.6E-01 |
| 3.6 | 450.320 | Glycohyodeoxycholic acid | 2b |  |  | 84 |  | POS | [M+H]+ | 6 | 28 | 5.3E-01 | 9.1E-01 |
| 1.6 | 453.209 | (1H)-Azulenone, 6-(beta-D-glucopyranosyloxy)-4,5,6,7,8,8a-hexahydro-5-hydroxy-5-(1-hydroxy-1-methylethyl)-3,8-dimethyl- | 2b |  |  | 83 |  | POS | [M+Na]+ | 6 | 105 | 8.8E-01 | 9.7E-01 |
| 6.6 | 454.292 | 1-Tridecanoyl-2-hydroxy-sn-glycero-3-phosphocholine | 2b |  |  | 75 |  | POS | [M+H]+ | 3 | 211 | 2.1E-01 | 9.1E-01 |
| 3.2 | 459.194 | 3,4-Dimethoxy-N-[(2S)-2-methyl-3-oxo-4-(2-phenylethyl)-5H-1,4-benzoxazepin-7-yl]benzamide | 3 |  |  |  | 48 | NEG | [M-H]- | 15 | 66 | 5.4E-02 | 6.6E-01 |
| 5.1 | 466.315 | Glycohyocholic acid | 2b |  |  | 81 |  | POS | [M+H]+ | 26 | 55 | 1.6E-01 | 9.1E-01 |
| 6.5 | 482.323 | 1-Pentadecanoyl-2-hydroxy-sn-glycero-3-phosphocholine | 2b |  |  | 76 |  | POS | [M+H]+ | 8 | 272 | 1.1E-01 | 8.6E-01 |
| 5.2 | 488.297 | Glycocholate | 2b |  |  | 82 |  | POS | [M+Na]+ | 4 | 69 | 6.4E-01 | 9.3E-01 |
| 0.9 | 492.475 | *N*-(1,3-dihydroxyoctadec-4-en-2-yl)tetradecanamide | 2b |  |  | 81 |  | POS | [M+H-H2O]- | 22 | 5 | 3.6E-01 | 9.1E-01 |
| 6.5 | 496.339 | PC(16:0/0:0) | 2b |  |  | 85 | 99 | POS | [M+H]+ | 7 | 16164 | 4.6E-01 | 9.1E-01 |
| 6.2 | 500.303 | Tauroursodeoxycholic acid | 2b |  |  | 86 |  | POS | [M+H]+ | 23 | 130 | 5.1E-01 | 9.1E-01 |
| 6.8 | 514.283 | Taurallocholic acid | 2b |  |  | 95 |  | NEG | [M-H]- | 1 | 4637 | 8.7E-03 | 2.3E-01 |
| 6.9 | 516.298 | Taurocholic acid | 2b |  |  | 86 | 99 | POS | [M+H]+ | 13 | 89 | 7.9E-01 | 9.4E-01 |
| 6.2 | 517.328 | Tauroursodeoxycholic acid | 2b |  |  | 86 |  | POS | [M+NH4]+ | 21 | 16 | 5.8E-01 | 9.3E-01 |
| 6.5 | 520.339 | LPC 18:2 | 2b |  |  | 72 |  | POS | [M+H]+ | 1 | 5154 | 3.3E-01 | 9.1E-01 |
| 6.5 | 526.289 | LysoPE(22:5(7Z,10Z,13Z,16Z,19Z)/0:0) | 3 |  |  |  | 99 | NEG | [M-H]- | 11 | 979 | 2.9E-02 | 5.5E-01 |
| 11.5 | 527.158 | 2-[3-[2-(1,3-benzodioxol-5-yl)-7-methoxy-1-benzofuran-5-yl]-3-hydroxypropoxy]-6-(hydroxymethyl)oxane-3,4,5-triol | 2b |  |  | 75 |  | POS | [M+Na]+ | 6 | 366 | 7.7E-01 | 9.4E-01 |
| 6.9 | 533.325 | Taurocholic acid | 2b |  |  | 85 |  | POS | [M+NH4]+ | 23 | 58 | 9.4E-01 | 9.9E-01 |
| 0.8 | 538.518 | Ceramide (18:1/16:0) | 2b |  |  | 79 |  | POS | [M+H]+ | 5 | 19 | 7.9E-01 | 9.4E-01 |
| 6.4 | 562.326 | LysoPC(0:0/18:0) | 2b |  |  | 82 | 96 | POS | [M+K]+ | 5 | 3132 | 7.4E-01 | 9.3E-01 |
| 6.4 | 570.350 | LysoPC(0:0/22:0) | 3 |  |  |  | 89 | POS | [M+H]+ | 10 | 344 | 5.1E-02 | 6.6E-01 |
| 6.4 | 587.330 | Inuline | 3 |  |  |  | 55 | POS | [M+H]+ | 8 | 23 | 7.2E-01 | 9.3E-01 |
| 6.4 | 640.254 | 11-[(1,3-dihydroxypropan-2-yl)oxy]-2-[4-hydroxy-3-(2-hydroxyethoxy)phenyl]-6-[(1H-indol-5-yl)methyl]-2H,3H,4H,5H,6H-phenanthro[2,1-b]pyran-3,8-diol | 3 |  |  |  | 41 | POS | [M+H]+ | 14 | 22 | 2.0E-01 | 9.1E-01 |
| 10.4 | 653.280 | 9a-Formyl-3a,5a-dihydroxy-11a-methyl-1-(5-oxo-2,5-dihydrofuran-3-yl)-7-[(3,4,5-trihydroxy-6-methyloxan-2-yl)oxy]-hexadecahydro-1H-cyclopenta[a]phenanthren-2-yl acetate | 3 |  |  |  | 69 | NEG | [M-H]- | 25 | 17 | 6.9E-01 | 9.3E-01 |
| 0.6 | 663.453 | 5-(1,2,4a,5-tetramethyl-7-oxo-3,4,8,8a-tetrahydro-2H-naphthalen-1-yl)-3-methylpentanoic acid | 2b |  |  | 73 |  | POS | [2M+Na]+ | 12 | 233 | 1.0E+00 | 1.0E+00 |
| 5.7 | 736.488 | PE(18:3(6Z,9Z,12Z)/18:2(9Z,12Z)) | 2b |  |  | 100 |  | NEG | [M-H]- | 20 | 569 | 8.7E-03 | 2.3E-01 |
| 5.5 | 780.551 | PC(18:4(6Z,9Z,12Z,15Z)/18:1(11Z)) | 3 |  |  |  | 98 | POS | [M+H]+ | 6 | 5930 | 8.2E-03 | 2.3E-01 |
| 6.0 | 795.560 | [(2E,4E,6E,8E,10E,12E,14E,16E)-17-[(1S,4R)-1,4-Dihydroxy-2,6,6-trimethylcyclohex-2-en-1-yl]-2-[2-[(4R)-4-hydroxy-2,6,6-trimethylcyclohexen-1-yl]acetyl]-6,11,15-trimethylheptadeca-2,4,6,8,10,12,14,16-octaenyl] (E)-dodec-2-enoate | 3 |  |  |  | 51 | NEG | [M-H]- | 12 | 29 | 1.3E-01 | 8.6E-01 |
| 0.4 | 850.782 | TAG(50:1) | 2b |  |  | 73 |  | POS | [M+NH4]+ | 10 | 3 | 9.9E-01 | 1.0E+00 |

**Supplemental Table 3. Labeled yeast amino acid CV values.**

| **Positive (+)** | **Labeled Yeast** | | | **Spiked Pooled Samples** | | |
| --- | --- | --- | --- | --- | --- | --- |
| Labeled Amino Acid | MEAN | ST DEV | CV | MEAN | ST DEV | CV |
| Glutamate | 4910625 | 273428.8 | 5.6 | 2592.4 | 183.4 | 7.1 |
| Methionine | 20650.3 | 172.2 | 0.8 | 452.3 | 4.2 | 0.9 |
| Phenylalanine | 76411.8 | 5788.3 | 7.6 | 7512.1 | 1228.5 | 16.4 |
| Proline | 1532608 | 101478.5 | 6.6 | 2370.7 | 241.6 | 10.2 |
| Serine | 110778.1 | 2725.4 | 2.5 | 7265.2 | 610.5 | 8.4 |
| Valine | 133035.7 | 5123.6 | 3.9 | 8676.5 | 951.9 | 11 |
| AVERAGE |  |  | 4.5 |  |  | 9 |
|  |  |  |  |  |  |  |
| **Negative (-)** | **Labeled Yeast** | | | **Spiked Pooled Samples** | | |
| Labeled Amino Acid | MEAN | ST DEV | CV | MEAN | ST DEV | CV |
| Asparagine | 212024.2 | 6039.2 | 2.8 | 708.4 | 89.4 | 12.6 |
| Aspartate | 2205421 | 33169.6 | 1.5 | 14866.9 | 347.7 | 2.3 |
| Glutamine | 2737371 | 62781.3 | 2.3 | 2700 | 161.2 | 6 |
| Threonine | 149169 | 12460.6 | 8.4 | 2380.6 | 457.2 | 19.2 |
| AVERAGE |  |  | 3.7 |  |  | 10 |

**Supplemental Table 4. Quality control samples for selected annotated features with associated CV values.**

|  | NEG |  |  |  |  |  |  |  |
| --- | --- | --- | --- | --- | --- | --- | --- | --- |
| RT | *m/z* | Metabolite name | QC1 | QC2 | QC3 | QC4 | QC5 | CV |
| 8.4 | 101.024 | Propionylformic acid | 24353 | 26295 | 32505 | 26806 | 27389 | 11.1 |
| 1.1 | 103.040 | Acetonic Acid | 11411 | 11493 | 11744 | 11137 | 10952 | 2.7 |
| 9.6 | 104.035 | Serine | 6813 | 6475 | 6536 | 6210 | 6141 | 4.2 |
| 7.8 | 114.055 | L-Proline | 34941 | 37071 | 36306 | 36614 | 34258 | 3.3 |
| 1.7 | 117.019 | Succinic acid | 20029 | 22251 | 20738 | 18119 | 26462 | 14.6 |
| 9.0 | 118.050 | alpha-Methyl-DL-serine | 9037 | 9117 | 8689 | 9303 | 9494 | 3.3 |
| 9.6 | 127.051 | Thymine | 14961 | 13876 | 14244 | 14074 | 15370 | 4.4 |
| 6.9 | 130.086 | Leucine | 936 | 1123 | 810 | 708 |  | 20.0 |
| 9.8 | 131.045 | Asparagine | 9442 | 9999 | 9502 | 10423 | 9326 | 4.7 |
| 5.9 | 135.031 | Allopurinol | 3667302 | 3747173 | 3816530 | 3811894 | 3739909 | 1.6 |
| 1.9 | 137.024 | 3-Hydroxybenzoic acid | 198137 | 301489 | 291758 | 283572 | 212648 | 18.7 |
| 9.6 | 145.061 | Glutamine | 52864 | 49996 | 50065 | 49095 | 55148 | 4.9 |
| 5.1 | 149.045 | Ribose | 13400 | 13067 | 13692 | 13819 | 12849 | 3.1 |
| 6.4 | 151.026 | Xanthine | 3578570 | 3655846 | 3699270 | 3613030 | 3860068 | 3.0 |
| 10.3 | 161.045 | Hydroxymethylglutaric acid | 275629 | 278852 | 284024 | 274985 | 276410 | 1.3 |
| 7.9 | 179.055 | Glucose | 49188 | 52105 | 54639 | 57020 | 45571 | 8.7 |
| 7.9 | 180.066 | Tyrosine | 145052 | 150351 | 155025 | 151901 | 152819 | 2.5 |
| 4.9 | 182.045 | 4-Pyridoxate | 80536 | 58423 | 58349 | 70334 | 86194 | 17.9 |
| 4.8 | 207.012 | 2-Naphthalenesulfonic acid | 178317 | 197344 | 205853 | 181751 | 229369 | 10.4 |
| 8.9 | 211.082 | Pyocyanin | 204329 | 197878 | 197362 | 184039 | 178866 | 5.5 |
| 1.9 | 216.981 | 5-Sulfosalicylic acid | 822564 | 1212227 | 1233067 | 1193060 | 886865 | 18.5 |
| 1.6 | 220.064 | N-lactoyl-Methionine | 30954 | 36526 | 35911 | 33775 | 42148 | 11.5 |
| 7.4 | 261.039 | Mannitol 1-phosphate | 12978 | 13549 | 13434 | 12880 | 13168 | 2.2 |
| 6.0 | 267.072 | Inosine | 44214 | 45353 | 42928 | 45824 | 44464 | 2.5 |
| 1.5 | 273.169 | 3-Hydroxytetradecanedioic acid | 41655 | 55263 | 49111 | 48784 | 55979 | 11.6 |
| 7.9 | 282.083 | Guanosine | 6687 | 6674 | 7004 | 7260 | 6704 | 3.8 |
| 7.1 | 283.067 | Acacetin | 133847 | 131213 | 130415 | 133936 | 145410 | 4.5 |
| 4.5 | 291.199 | (2'E,4'Z,7'Z,8E)-Colnelenic acid | 169875 | 172149 | 157615 | 194217 | 154533 | 9.2 |
| 10.3 | 341.107 | Trehalose | 9801 | 9977 | 9708 | 9949 | 10014 | 1.3 |
| 1.0 | 358.238 | 11-(2H-1,3-Benzodioxol-5-yl)-N-(2-methylpropyl)undec-10-enamide | 11805 | 12500 | 11235 | 9777 | 14550 | 14.7 |
| 6.9 | 375.129 | Riboflavin | 51281 | 50382 | 51958 | 50781 | 50182 | 1.4 |
| 6.9 | 411.106 | Riboflavin Cl- | 81592 | 85031 | 84186 | 86181 | 84132 | 2.0 |
| 5.2 | 414.095 | 2-(Glutathione-S-yl)-hydroquinone | 275249 | 276832 | 257665 | 255281 | 321151 | 9.5 |
| 1.0 | 414.226 | Altercrasin A | 57304 | 61467 | 53805 | 49606 | 67170 | 11.7 |
| 5.5 | 447.132 | Glycitin | 49347 | 56477 | 41880 | 48479 | 65186 | 17.0 |
| 3.0 | 448.305 | Chenodeoxyglycocholic acid | 374823 | 378488 | 370668 | 354660 | 430402 | 7.5 |
| 3.2 | 459.194 | 3,4-Dimethoxy-N-[(2S)-2-methyl-3-oxo-4-(2-phenylethyl)-5H-1,4-benzoxazepin-7-yl]benzamide | 22817 | 23406 | 27297 | 17723 | 22456 | 15.0 |
| 6.8 | 514.283 | Taurallocholic acid | 1008480 | 1034586 | 1006473 | 1012814 | 1026853 | 1.2 |
| 6.5 | 526.289 | LysoPE(22:5(7Z,10Z,13Z,16Z,19Z)/0:0) | 35109 | 42345 | 42156 | 44633 | 35091 | 11.2 |
| 5.7 | 736.488 | PE(18:3(6Z,9Z,12Z)/18:2(9Z,12Z)) | 383828 | 419573 | 242297 | 399144 | 404058 | 19.6 |
|  |  |  |  |  |  |  | Average | 8.1 |
|  | POS |  |  |  |  |  |  |  |
| RT | *m/z* | Metabolite name | QC1 | QC2 | QC3 | QC4 | QC5 | CV |
| 9.0 | 102.055 | DL-3-Aminoisobutyric acid | 11852 | 12883 | 10813 | 12564 | 11663 | 6.8 |
| 5.0 | 104.107 | Choline | 43356644 | 40251024 | 37663256 | 39201328 | 38139340 | 5.7 |
| 9.5 | 106.049 | Serine | 5227 | 6064 | 5279 | 5590 | 5162 | 6.8 |
| 2.5 | 113.034 | Uracil | 741940 | 791887 | 820649 | 827674 | 788953 | 4.3 |
| 6.6 | 114.066 | Creatinine | 38643 | 48516 | 35996 | 36527 | 36875 | 13.3 |
| 7.8 | 116.070 | Proline | 281752 | 264537 | 287018 | 272457 | 255680 | 4.7 |
| 4.3 | 118.085 | 4-Methylmorpholine-N-oxide | 74467 | 96281 | 109539 | 90074 | 93742 | 13.6 |
| 7.0 | 118.086 | Valine | 15685013 | 12314992 | 11913969 | 11484300 | 10974891 | 14.9 |
| 6.8 | 120.080 | Indoline | 1984197 | 2130827 | 1916917 | 1870867 | 1828912 | 6.1 |
| 1.9 | 122.096 | 2,4,6-Trimethylpyridine | 806883 | 1000118 | 995584 | 1071811 | 1035306 | 10.4 |
| 8.4 | 123.055 | Niacinamide | 2458346 | 2530700 | 2483509 | 2399690 | 2570790 | 2.6 |
| 1.3 | 124.039 | Nicotinic acid | 550742 | 331448 | 515310 | 443089 | 457088 | 18.2 |
| 8.2 | 126.021 | Taurine | 25016 | 28512 | 23854 | 21903 | 22224 | 11.0 |
| 1.1 | 129.075 | Pyroglutamate | 29847 | 23510 | 25448 | 24050 | 25000 | 9.8 |
| 9.5 | 130.050 | delta-Aminolevulinic acid | 58975 | 67288 | 70979 | 69088 | 67140 | 6.9 |
| 7.3 | 132.102 | Leucine/Isoleucine | 64163 | 74856 | 56177 | 58396 | 54759 | 13.3 |
| 9.7 | 133.060 | Asparagine | 4893 | 5405 | 6217 | 4557 | 3868 | 17.7 |
| 6.7 | 133.104 | L-Ornithine | 37609 | 31516 | 31292 | 26141 | 31395 | 12.9 |
| 5.7 | 136.061 | Adenine | 121843 | 116177 | 95192 | 86519 | 93657 | 15.0 |
| 5.7 | 137.046 | Allopurinol | 52208448 | 51622624 | 54131460 | 55424228 | 54682636 | 3.0 |
| 12.8 | 144.047 | 4-Hydroxyquinoline | 46135 | 53777 | 39446 | 37265 | 39670 | 15.6 |
| 7.1 | 146.059 | 3-Formylindole | 30048 | 37122 | 44216 | 50075 | 47448 | 19.5 |
| 5.2 | 146.117 | Acetylcholine | 121112 | 131814 | 112997 | 141254 | 145862 | 10.5 |
| 9.5 | 147.076 | L-Glutamine | 28028 | 24856 | 25877 | 30277 | 25980 | 8.0 |
| 7.5 | 150.058 | Methionine | 86513 | 86076 | 72597 | 70808 | 66573 | 12.0 |
| 7.7 | 152.056 | Acenapthylene | 15216 | 15427 | 12686 | 14406 | 14361 | 7.5 |
| 6.2 | 153.041 | Xanthine (not validated) | 3988246 | 3966374 | 4324364 | 3907769 | 4006138 | 4.1 |
| 13.6 | 156.076 | L-Histidine | 8978 | 9350 | 8868 | 8347 | 7441 | 8.6 |
| 10.9 | 162.075 | 2-Aminoadipic acid | 4777 | 5482 | 4567 | 5658 | 4596 | 10.3 |
| 6.6 | 162.112 | L-Carnitine | 144490 | 129893 | 109923 | 125692 | 121765 | 10.0 |
| 7.1 | 166.086 | Phenylalanine | 511110 | 487964 | 479147 | 461332 | 426453 | 6.7 |
| 7.2 | 175.116 | L-Arginine | 4023 | 4177 | 5376 | 4602 | 4448 | 11.6 |
| 7.9 | 182.081 | Tyrosine | 64836 | 81539 | 86600 | 82703 | 80571 | 10.6 |
| 0.7 | 201.163 | Isolongifolene, 4,5,9,10-dehydro- | 29092 | 31121 | 29346 | 26796 | 36058 | 11.4 |
| 1.9 | 212.118 | 1,3-Diphenylguanidine | 464896 | 489296 | 418053 | 517266 | 466280 | 7.8 |
| 2.5 | 218.138 | Propionylcarnitine | 39839 | 40875 | 48864 | 55808 | 36254 | 17.8 |
| 2.0 | 232.154 | Butyryl carnitine | 34010 | 42114 | 34200 | 36133 | 42156 | 10.9 |
| 8.8 | 244.093 | Cytidine | 79235 | 84239 | 80129 | 73022 | 71449 | 6.8 |
| 10.1 | 258.110 | 5-Methylcytidine | 55020 | 50476 | 55112 | 55163 | 51897 | 4.1 |
| 36.9 | 268.103 | Adenosine | 220201 | 227068 | 295248 | 280381 | 281432 | 13.3 |
| 5.7 | 298.096 | 5'-S-Methyl-5'-thioadenosine | 198896 | 194104 | 178624 | 193202 | 170817 | 6.3 |
| 1.0 | 299.200 | 3,4-Didehydroretinoic acid | 53439 | 53517 | 67051 | 58626 | 53252 | 10.4 |
| 4.5 | 337.272 | Isolinderanolide | 113292 | 98149 | 114760 | 106276 | 83257 | 12.5 |
| 6.8 | 377.145 | Riboflavin | 43759 | 47487 | 47043 | 47788 | 42730 | 5.1 |
| 1.0 | 399.146 | S-(5'-Adenosyl)-L-methionine | 23301 | 20540 | 28531 | 30267 | 26632 | 15.2 |
| 6.5 | 496.339 | PC(16:0/0:0) | 11844732 | 10536915 | 10229182 | 10442106 | 9902360 | 7.0 |
| 6.9 | 516.298 | Taurocholic acid | 101031 | 71425 | 83677 | 95280 | 87436 | 12.9 |
| 6.4 | 640.254 | Unknown Feature | 15324 | 18717 | 15911 | 12274 | 11747 | 19.3 |
| 5.5 | 780.551 | PC(18:4(6Z,9Z,12Z,15Z)/18:1(11Z)) | 4952114 | 5309093 | 4994954 | 4790562 | 4782601 | 4.3 |
|  |  |  |  |  |  |  | Average | 10.0 |

**Supplemental Table 5. Statistically significant untargeted features with ANOVA *P-*values and quality control CVs.**

| **RT** | **m/z** | **ANOVA *P-*value** | **Mode** | **Adduct** | **Signal/Noise** | **Average Area** | **QC CV** |
| --- | --- | --- | --- | --- | --- | --- | --- |
| 1.8 | 105.044 | 4.9E-02 | POS | [M+H]+ | 93 | 4210 | 6.4 |
| 1.0 | 109.064 | 6.7E-03 | POS | [M+H]+ | 12 | 721 | 8.4 |
| 0.7 | 113.096 | 4.8E-02 | POS | [M+H]+ | 29 | 1105 | 2.9 |
| 12.4 | 118.086 | 3.9E-02 | POS | [M+H]+ | 9 | 649 | 8.1 |
| 9.8 | 118.087 | 4.5E-02 | POS | [M+H]+ | 77 | 1141 | 5.0 |
| 5.7 | 120.019 | 4.5E-02 | POS | [M+H]+ | 272 | 13772 | 9.2 |
| 2.0 | 120.101 | 4.7E-02 | POS | [M+H]+ | 200 | 8519 | 11.3 |
| 2.4 | 121.028 | 4.4E-02 | POS | [M+H]+ | 40 | 543 | 13.7 |
| 1.7 | 122.096 | 2.2E-02 | POS | [M+H]+ | 790 | 24348 | 10.4 |
| 0.9 | 131.162 | 4.1E-02 | POS | [M+H]+ | 9 | 552 | 16.1 |
| 0.9 | 133.085 | 1.1E-02 | POS | [M+H]+ | 21 | 1065 | 14.0 |
| 8.9 | 134.032 | 2.7E-02 | POS | [M+H]+ | 21 | 935 | 4.6 |
| 5.5 | 139.086 | 1.7E-03 | POS | [M+H]+ | 13 | 123 | 27.7 |
| 8.9 | 140.068 | 2.3E-02 | POS | [M+H]+ | 56 | 2922 | 7.2 |
| 11.0 | 141.078 | 5.9E-03 | POS | [M+H]+ | 37 | 46 | 8.3 |
| 1.2 | 143.106 | 3.1E-02 | POS | [M+H]+ | 23 | 1151 | 3.8 |
| 1.3 | 144.982 | 1.3E-02 | POS | [M+H]+ | 10 | 298 | 22.6 |
| 6.0 | 148.042 | 1.8E-02 | POS | [M+H]+ | 91 | 4062 | 4.2 |
| 4.8 | 148.087 | 2.6E-02 | POS | [M+H]+ | 8 | 388 | 11.1 |
| 0.2 | 148.087 | 3.5E-02 | POS | [M+H]+ | 6 | 710 | 4.3 |
| 1.3 | 151.096 | 4.4E-02 | POS | [M+H]+ | 19 | 1287 | 14.9 |
| 7.5 | 155.075 | 4.7E-02 | POS | [M+H]+ | 22 | 43 | 3.4 |
| 3.9 | 158.154 | 2.5E-02 | POS | [M+H]+ | 70 | 4081 | 11.7 |
| 12.5 | 164.112 | 3.8E-02 | POS | [M+H]+ | 163 | 74 | 6.2 |
| 4.7 | 170.096 | 1.6E-02 | POS | [M+H]+ | 4 | 591 | 14.0 |
| 1.8 | 171.116 | 4.2E-02 | POS | [M+H]+ | 14 | 549 | 15.1 |
| 5.2 | 172.133 | 8.0E-03 | POS | [M+H]+ | 27 | 1691 | 9.3 |
| 4.2 | 173.081 | 2.3E-02 | POS | [M+H]+ | 10 | 596 | 11.3 |
| 5.9 | 176.117 | 1.6E-02 | POS | [M+H]+ | 18 | 1002 | 10.4 |
| 9.1 | 182.962 | 3.8E-02 | POS | [M+H]+ | 8 | 94 | 12.7 |
| 13.8 | 182.963 | 1.2E-02 | POS | [M+H]+ | 5 | 305 | 4.2 |
| 1.0 | 191.179 | 3.9E-02 | POS | [M+H]+ | 15 | 691 | 5.1 |
| 5.1 | 209.123 | 3.1E-03 | POS | [M+H]+ | 4 | 141 | 22.8 |
| 2.5 | 218.138 | 8.5E-03 | POS | [M+H]+ | 78 | 1821 | 15.2 |
| 13.8 | 220.919 | 1.4E-02 | POS | [M+H]+ | 4 | 228 | 9.8 |
| 2.0 | 232.154 | 6.2E-04 | POS | [M+H]+ | 103 | 2758 | 16.6 |
| 1.3 | 234.112 | 1.3E-02 | POS | [M+Na]+ | 9 | 575 | 14.4 |
| 5.0 | 243.118 | 1.5E-02 | POS | [M+H]+ | 13 | 582 | 14.2 |
| 2.6 | 244.189 | 2.1E-02 | POS | [M+H]+ | 31 | 1764 | 0.6 |
| 4.7 | 263.084 | 3.8E-02 | POS | [M+H]+ | 15 | 779 | 9.4 |
| 1.0 | 263.151 | 1.3E-02 | POS | [M+H]+ | 34 | 993 | 7.5 |
| 1.1 | 266.110 | 8.0E-03 | POS | [M+H]+ | 15 | 1091 | 11.8 |
| 13.8 | 270.838 | 4.6E-02 | POS | [M+Na]+ | 4 | 226 | 21.0 |
| 13.8 | 270.858 | 3.7E-03 | POS | [M+H]+ | 3 | 41 | 16.3 |
| 8.0 | 272.061 | 4.2E-02 | POS | [M+H]+ | 6 | 240 | 8.5 |
| 1.0 | 275.199 | 3.6E-02 | POS | [M+H]+ | 20 | 999 | 10.7 |
| 5.2 | 276.179 | 2.4E-03 | POS | [M+H]+ | 21 | 419 | 4.9 |
| 14.0 | 281.051 | 4.5E-02 | POS | [M+H]+ | 3 | 303 | 6.2 |
| 5.3 | 281.121 | 3.6E-02 | POS | [M+H]+ | 3 | 160 | 15.9 |
| 1.0 | 281.174 | 4.4E-02 | POS | [M+H]+ | 20 | 997 | 9.1 |
| 1.0 | 297.241 | 1.6E-02 | POS | [M+H]+ | 26 | 1380 | 7.5 |
| 1.8 | 302.270 | 1.8E-02 | POS | [M+H]+ | 7 | 143 | 5.4 |
| 9.4 | 303.178 | 3.5E-02 | POS | [M+H]+ | 15 | 26 | 9.1 |
| 1.0 | 313.237 | 4.4E-02 | POS | [M+H]+ | 12 | 543 | 1.3 |
| 9.1 | 317.121 | 4.7E-03 | POS | [M+H]+ | 47 | 91 | 3.1 |
| 1.0 | 317.210 | 4.6E-02 | POS | [M+Na]+ | 31 | 1590 | 8.1 |
| 0.7 | 319.263 | 2.3E-02 | POS | [M+H]+ | 35 | 1730 | 4.8 |
| 1.0 | 327.224 | 1.0E-02 | POS | [M+Na]+ | 20 | 1193 | 12.6 |
| 1.0 | 329.185 | 4.2E-02 | POS | [M+Na]+ | 12 | 609 | 11.2 |
| 1.0 | 331.256 | 4.0E-02 | POS | [M+H]+ | 10 | 616 | 11.7 |
| 8.8 | 334.062 | 2.3E-02 | POS | [M+H]+ | 26 | 1068 | 4.3 |
| 1.0 | 335.219 | 4.5E-02 | POS | [M+H]+ | 40 | 1984 | 13.1 |
| 4.5 | 335.257 | 3.8E-02 | POS | [M+H]+ | 21 | 1043 | 12.2 |
| 4.1 | 337.121 | 4.9E-02 | POS | [M+H]+ | 15 | 898 | 9.2 |
| 4.4 | 339.288 | 1.9E-02 | POS | [M+H]+ | 60 | 3110 | 15.4 |
| 4.5 | 339.288 | 1.9E-02 | POS | [M+H]+ | 57 | 3110 | 15.4 |
| 13.8 | 340.821 | 4.5E-02 | POS | [M+H]+ | 5 | 151 | 19.8 |
| 1.0 | 341.216 | 3.5E-02 | POS | [M+H]+ | 17 | 646 | 9.8 |
| 3.6 | 341.242 | 4.1E-02 | POS | [M+H]+ | 29 | 1832 | 5.6 |
| 13.5 | 352.856 | 1.7E-02 | POS | [M+H]+ | 10 | 235 | 16.1 |
| 6.6 | 357.214 | 4.2E-02 | POS | [M+2H]2+ | 19 | 1150 | 14.7 |
| 4.3 | 361.272 | 2.9E-02 | POS | [M+H]+ | 21 | 876 | 10.7 |
| 4.4 | 361.274 | 3.6E-03 | POS | [M+H]+ | 18 | 938 | 9.1 |
| 0.8 | 362.268 | 1.9E-02 | POS | [M+Na]+ | 35 | 1561 | 10.6 |
| 4.4 | 363.287 | 2.7E-02 | POS | [M+H]+ | 9 | 497 | 7.1 |
| 1.6 | 370.275 | 3.1E-02 | POS | [M+H]+ | 38 | 716 | 9.8 |
| 13.2 | 375.872 | 2.7E-02 | POS | [M+H]+ | 22 | 274 | 11.8 |
| 13.8 | 376.820 | 3.4E-02 | POS | [M+Na]+ | 10 | 168 | 12.8 |
| 0.9 | 377.269 | 3.8E-02 | POS | [M+H]+ | 11 | 587 | 3.0 |
| 1.2 | 382.154 | 4.1E-02 | POS | [M+H]+ | 22 | 969 | 8.8 |
| 1.0 | 388.286 | 2.2E-02 | POS | [M+H]+ | 23 | 1045 | 9.2 |
| 1.8 | 395.165 | 4.4E-02 | POS | [M+H]+ | 5 | 97 | 15.3 |
| 1.1 | 395.286 | 2.1E-02 | POS | [M+H]+ | 26 | 936 | 25.8 |
| 1.0 | 398.238 | 2.3E-06 | POS | [M+H]+ | 27 | 588 | 9.9 |
| 13.7 | 399.803 | 3.8E-02 | POS | [M+H]+ | 5 | 193 | 13.0 |
| 12.4 | 399.873 | 3.9E-02 | POS | [M+H]+ | 3 | 50 | 10.1 |
| 0.9 | 400.248 | 3.3E-02 | POS | [M+H]+ | 13 | 793 | 4.9 |
| 5.2 | 400.281 | 3.5E-02 | POS | [M+H]+ | 4 | 178 | 19.5 |
| 1.0 | 411.285 | 4.9E-02 | POS | [M+H]+ | 10 | 738 | 16.6 |
| 4.9 | 416.110 | 4.1E-02 | POS | [M+H]+ | 249 | 13211 | 5.8 |
| 1.0 | 417.266 | 5.0E-02 | POS | [M+H]+ | 14 | 797 | 11.4 |
| 1.1 | 438.357 | 3.2E-02 | POS | [M+H]+ | 23 | 758 | 14.9 |
| 4.9 | 440.091 | 3.8E-02 | POS | [M+Na]+ | 15 | 709 | 8.6 |
| 0.8 | 441.360 | 4.6E-02 | POS | [M+H]+ | 6 | 425 | 5.0 |
| 1.0 | 447.331 | 3.9E-03 | POS | [M+H]+ | 8 | 397 | 18.8 |
| 6.8 | 453.055 | 8.2E-03 | POS | [M+H]+ | 4 | 170 | 22.6 |
| 1.9 | 460.256 | 3.7E-02 | POS | [M+H]+ | 22 | 1064 | 4.0 |
| 6.0 | 461.274 | 2.9E-02 | POS | [M+H]+ | 3 | 147 | 15.8 |
| 4.7 | 472.311 | 4.8E-02 | POS | [M+H]+ | 7 | 939 | 11.3 |
| 5.1 | 472.311 | 7.1E-03 | POS | [M+H]+ | 10 | 641 | 9.8 |
| 1.0 | 489.277 | 1.7E-02 | POS | [M+H]+ | 12 | 618 | 11.6 |
| 1.0 | 489.284 | 2.8E-02 | POS | [M+H]+ | 10 | 594 | 11.2 |
| 6.5 | 501.278 | 4.4E-02 | POS | [M+H]+ | 13 | 1235 | 11.6 |
| 6.5 | 508.338 | 4.9E-02 | POS | [M+H]+ | 51 | 2143 | 8.6 |
| 7.9 | 513.352 | 4.6E-02 | POS | [M+H]+ | 4 | 203 | 11.5 |
| 4.4 | 515.409 | 9.9E-03 | POS | [M+H]+ | 9 | 500 | 17.6 |
| 14.5 | 515.412 | 4.3E-02 | POS | [M+H]+ | 14 | 1620 | 8.4 |
| 4.4 | 517.364 | 1.8E-02 | POS | [M+H]+ | 6 | 250 | 13.7 |
| 14.7 | 519.512 | 2.3E-02 | POS | [M+H]+ | 5 | 240 | 2.5 |
| 6.2 | 522.285 | 2.3E-02 | POS | [M+Na]+ | 31 | 1071 | 16.0 |
| 5.0 | 526.430 | 1.3E-02 | POS | [M+H]+ | 14 | 639 | 12.8 |
| 8.8 | 531.142 | 4.7E-02 | POS | [M+H]+ | 24 | 1016 | 16.8 |
| 1.0 | 533.417 | 2.1E-02 | POS | [M+H]+ | 5 | 235 | 16.4 |
| 7.6 | 547.873 | 4.1E-02 | POS | [M+H]+ | 3 | 165 | 3.5 |
| 7.7 | 560.597 | 1.1E-02 | POS | [M+H]+ | 4 | 111 | 2.5 |
| 4.9 | 571.255 | 1.3E-02 | POS | [M+H]+ | 6 | 348 | 6.2 |
| 13.5 | 573.685 | 4.3E-02 | POS | [M+H]+ | 3 | 199 | 17.0 |
| 7.6 | 576.486 | 2.4E-02 | POS | [M+H]+ | 3 | 74 | 11.3 |
| 1.0 | 582.361 | 2.7E-02 | POS | [M+H]+ | 5 | 227 | 13.1 |
| 1.0 | 583.359 | 4.4E-02 | POS | [M+Na]+ | 9 | 327 | 8.3 |
| 6.6 | 594.209 | 3.8E-02 | POS | [M+H]+ | 39 | 2283 | 9.1 |
| 13.8 | 594.704 | 4.9E-02 | POS | [M+H]+ | 5 | 290 | 5.7 |
| 1.1 | 595.347 | 4.4E-02 | POS | [M+H]+ | 13 | 468 | 15.4 |
| 7.7 | 598.192 | 3.2E-02 | POS | [M+2H]2+ | 3 | 77 | 3.8 |
| 8.8 | 599.127 | 7.5E-03 | POS | [M+Na]+ | 11 | 364 | 6.9 |
| 13.5 | 606.685 | 3.2E-02 | POS | [M+H]+ | 7 | 342 | 7.8 |
| 6.3 | 610.313 | 4.0E-02 | POS | [M+H]+ | 69 | 2792 | 17.5 |
| 1.1 | 616.372 | 3.9E-02 | POS | [M+H]+ | 6 | 266 | 12.7 |
| 13.8 | 621.683 | 4.7E-02 | POS | [M+H]+ | 3 | 153 | 15.4 |
| 6.5 | 627.431 | 8.6E-03 | POS | [M+H]+ | 10 | 468 | 5.8 |
| 13.6 | 641.680 | 2.9E-02 | POS | [M+H]+ | 5 | 304 | 18.4 |
| 1.5 | 661.201 | 4.2E-02 | POS | [M+H]+ | 9 | 218 | 7.7 |
| 1.0 | 670.509 | 1.7E-02 | POS | [M+H]+ | 18 | 95 | 8.0 |
| 6.6 | 671.330 | 4.0E-02 | POS | [M+H]+ | 19 | 860 | 8.7 |
| 6.4 | 679.402 | 1.9E-02 | POS | [M+H]+ | 32 | 1676 | 11.8 |
| 5.6 | 700.488 | 4.2E-02 | POS | [M+H]+ | 6 | 184 | 3.9 |
| 1.0 | 707.435 | 3.2E-02 | POS | [M+H]+ | 4 | 252 | 10.0 |
| 1.0 | 710.414 | 2.9E-02 | POS | [M+H]+ | 3 | 129 | 23.3 |
| 14.7 | 712.643 | 2.1E-02 | POS | [M+H]+ | 7 | 359 | 5.4 |
| 8.9 | 713.117 | 4.4E-02 | POS | [M+H]+ | 4 | 180 | 14.3 |
| 1.0 | 713.373 | 3.2E-02 | POS | [M+Na]+ | 4 | 158 | 12.9 |
| 13.7 | 714.692 | 1.4E-02 | POS | [M+H]+ | 3 | 151 | 21.4 |
| 1.0 | 719.504 | 4.0E-02 | POS | [M+H]+ | 3 | 143 | 22.2 |
| 4.5 | 721.292 | 2.0E-02 | POS | [M+H]+ | 5 | 42 | 14.8 |
| 1.0 | 722.445 | 4.4E-02 | POS | [M+H]+ | 3 | 174 | 27.1 |
| 5.6 | 732.552 | 9.7E-03 | POS | [M+H]+ | 4070 | 248084 | 3.9 |
| 13.5 | 737.730 | 2.2E-02 | POS | [M+H]+ | 4 | 192 | 12.1 |
| 5.7 | 747.537 | 3.4E-03 | POS | [M+H]+ | 10 | 534 | 23.5 |
| 5.8 | 748.547 | 1.1E-02 | POS | [M+H]+ | 32 | 1291 | 7.5 |
| 8.7 | 764.388 | 3.0E-02 | POS | [M+H]+ | 12 | 355 | 11.7 |
| 4.8 | 766.533 | 7.4E-03 | POS | [M+H]+ | 16 | 537 | 24.0 |
| 14.5 | 780.552 | 4.5E-02 | POS | [M+H]+ | 10 | 437 | 18.6 |
| 4.5 | 787.364 | 3.4E-02 | POS | [M+H]+ | 6 | 141 | 17.7 |
| 4.4 | 801.381 | 2.9E-02 | POS | [M+H]+ | 5 | 127 | 16.0 |
| 8.2 | 802.533 | 1.8E-02 | POS | [M+H]+ | 11 | 435 | 14.4 |
| 6.3 | 804.090 | 3.7E-02 | POS | [M+H]+ | 80 | 3692 | 6.3 |
| 6.6 | 811.990 | 2.5E-02 | POS | [M+H]+ | 6 | 221 | 21.9 |
| 5.8 | 817.579 | 1.8E-02 | POS | [M+H]+ | 10 | 754 | 7.2 |
| 5.7 | 828.545 | 3.8E-02 | POS | [M+H]+ | 47 | 5212 | 7.7 |
| 7.6 | 828.810 | 4.8E-02 | POS | [M+H]+ | 5 | 261 | 16.3 |
| 0.4 | 848.768 | 4.8E-02 | POS | [M+H]+ | 3 | 167 | 14.8 |
| 6.6 | 860.288 | 1.8E-02 | POS | [M+H]+ | 33 | 1952 | 14.1 |
| 6.3 | 953.955 | 4.8E-02 | POS | [M+H]+ | 45 | 2103 | 18.8 |
| 3.1 | 100.003 | 1.0E-02 | NEG | [M-H]- | 13 | 6277 | 21.5 |
| 1.1 | 107.050 | 2.1E-02 | NEG | [M-H]- | 13 | 5882 | 11.2 |
| 1.0 | 107.053 | 1.3E-02 | NEG | [M-H]- | 17 | 5436 | 11.2 |
| 1.1 | 114.023 | 3.8E-02 | NEG | [M-H]- | 10 | 4974 | 23.4 |
| 1.1 | 130.086 | 3.2E-02 | NEG | [M-H]- | 15 | 9047 | 7.9 |
| 1.1 | 130.086 | 4.0E-02 | NEG | [M-H]- | 25 | 7002 | 10.2 |
| 4.1 | 146.965 | 3.5E-03 | NEG | [M-H]- | 15 | 28110 | 9.0 |
| 5.3 | 182.988 | 4.0E-02 | NEG | [M-H]- | 32 | 72944 | 6.4 |
| 1.2 | 186.045 | 3.3E-02 | NEG | [M-H]- | 59 | 65366 | 21.0 |
| 1.1 | 186.045 | 3.3E-02 | NEG | [M-H]- | 112 | 66938 | 21.2 |
| 1.2 | 187.041 | 4.3E-02 | NEG | [M-H]- | 233 | 271345 | 17.6 |
| 1.1 | 187.042 | 4.1E-02 | NEG | [M-H]- | 459 | 281239 | 18.0 |
| 1.1 | 188.045 | 1.5E-02 | NEG | [M-H]- | 35 | 21283 | 11.3 |
| 2.6 | 188.986 | 2.7E-03 | NEG | [M-H]- | 10 | 7358 | 28.5 |
| 3.1 | 194.045 | 5.0E-02 | NEG | [M-H]- | 24 | 22429 | 16.2 |
| 1.2 | 205.034 | 2.8E-02 | NEG | [M-H]- | 25 | 15359 | 9.7 |
| 1.2 | 205.035 | 8.7E-03 | NEG | [M-H]- | 17 | 14687 | 9.8 |
| 4.6 | 216.980 | 2.3E-02 | NEG | [M-H]- | 36 | 18235 | 27.6 |
| 4.6 | 216.981 | 2.2E-02 | NEG | [M-H]- | 12 | 19515 | 24.5 |
| 1.5 | 220.064 | 3.1E-02 | NEG | [M-H]- | 80 | 34550 | 14.5 |
| 6.4 | 224.958 | 3.2E-02 | NEG | [M-H]- | 15 | 15215 | 10.5 |
| 3.0 | 244.136 | 4.6E-02 | NEG | [M-H]- | 116 | 63826 | 22.8 |
| 1.4 | 254.981 | 3.3E-02 | NEG | [M-H]- | 1505 | 1945192 | 24.6 |
| 6.9 | 255.087 | 4.8E-02 | NEG | [M-H]- | 17 | 12900 | 4.8 |
| 6.6 | 256.058 | 2.9E-02 | NEG | [M-H]- | 20 | 3327 | 14.0 |
| 6.7 | 274.128 | 4.3E-02 | NEG | [M-H]- | 31 | 15731 | 4.6 |
| 1.1 | 285.133 | 3.5E-02 | NEG | [M-H]- | 16 | 14279 | 13.4 |
| 1.1 | 287.148 | 4.4E-02 | NEG | [M-H]- | 12 | 10392 | 23.3 |
| 4.5 | 291.199 | 2.6E-02 | NEG | [M-H]- | 163 | 241615 | 14.1 |
| 4.5 | 292.202 | 3.4E-02 | NEG | [M-H]- | 31 | 41764 | 12.8 |
| 3.0 | 295.129 | 3.4E-02 | NEG | [M-H]- | 265 | 257156 | 7.7 |
| 2.9 | 295.129 | 4.5E-02 | NEG | [M-H]- | 240 | 335846 | 9.1 |
| 4.6 | 298.978 | 1.8E-02 | NEG | [M-H]- | 13 | 5453 | 27.5 |
| 1.1 | 301.201 | 5.7E-04 | NEG | [M-H]- | 13 | 13058 | 8.2 |
| 1.2 | 301.201 | 3.1E-03 | NEG | [M-H]- | 13 | 13058 | 8.2 |
| 1.2 | 302.055 | 4.0E-02 | NEG | [M-H]- | 11 | 4777 | 10.5 |
| 2.5 | 302.127 | 2.5E-02 | NEG | [M-H]- | 10 | 13656 | 29.1 |
| 1.2 | 303.051 | 5.0E-02 | NEG | [M-H]- | 23 | 17961 | 15.6 |
| 4.4 | 305.214 | 4.0E-02 | NEG | [M-H]- | 141 | 83426 | 26.8 |
| 3.0 | 314.937 | 1.0E-02 | NEG | [M-H]- | 32 | 60693 | 16.1 |
| 1.1 | 317.104 | 4.2E-02 | NEG | [M-H]- | 14 | 8987 | 19.2 |
| 1.1 | 317.105 | 3.1E-02 | NEG | [M-H]- | 21 | 8776 | 23.3 |
| 6.5 | 330.698 | 1.3E-02 | NEG | [M-H]- | 192 | 82075 | 6.1 |
| 8.5 | 330.962 | 1.9E-02 | NEG | [M-H]- | 24 | 21200 | 14.1 |
| 5.8 | 333.092 | 1.1E-02 | NEG | [M-H]- | 22 | 11403 | 22.7 |
| 6.6 | 340.726 | 1.9E-02 | NEG | [M-H]- | 11 | 9365 | 7.1 |
| 6.5 | 340.726 | 4.0E-02 | NEG | [M-H]- | 12 | 9137 | 7.3 |
| 6.6 | 342.724 | 1.6E-02 | NEG | [M-H]- | 22 | 13032 | 18.0 |
| 6.8 | 356.999 | 4.4E-02 | NEG | [M-H]- | 16 | 8246 | 23.1 |
| 4.7 | 358.196 | 2.4E-02 | NEG | [M-H]- | 16 | 7060 | 12.6 |
| 5.9 | 374.167 | 4.7E-02 | NEG | [M-H]- | 29 | 2258 | 29.2 |
| 6.9 | 375.129 | 8.7E-04 | NEG | [M-H]- | 69 | 50831 | 7.1 |
| 6.9 | 375.129 | 3.5E-04 | NEG | [M-H]- | 77 | 48036 | 6.6 |
| 0.8 | 377.195 | 3.9E-02 | NEG | [M-H]- | 42 | 37036 | 26.7 |
| 3.1 | 384.197 | 1.7E-02 | NEG | [M-H]- | 51 | 25022 | 25.6 |
| 1.1 | 397.095 | 1.1E-07 | NEG | [M-H]- | 13 | 2481 | 20.4 |
| 0.8 | 398.232 | 5.2E-14 | NEG | [M-H]- | 187 | 63487 | 20.2 |
| 4.6 | 399.223 | 4.2E-02 | NEG | [M-H]- | 26 | 21579 | 13.3 |
| 1.1 | 399.237 | 1.9E-02 | NEG | [M-H]- | 11 | 5647 | 22.1 |
| 0.8 | 400.244 | 1.3E-04 | NEG | [M-H]- | 19 | 9567 | 19.9 |
| 6.9 | 411.105 | 7.4E-04 | NEG | [M-H]- | 103 | 80496 | 3.4 |
| 6.9 | 411.106 | 2.0E-03 | NEG | [M+Cl]- | 109 | 84448 | 4.3 |
| 6.5 | 412.619 | 2.5E-02 | NEG | [M-H]- | 45 | 9213 | 19.4 |
| 6.6 | 412.620 | 3.9E-02 | NEG | [M-H]- | 16 | 9349 | 18.6 |
| 6.9 | 413.103 | 1.6E-03 | NEG | [M-H]- | 37 | 28182 | 5.2 |
| 1.6 | 413.163 | 3.3E-02 | NEG | [M-H]- | 18 | 6602 | 21.6 |
| 5.2 | 414.095 | 5.2E-03 | NEG | [M-H]- | 456 | 280128 | 9.9 |
| 5.1 | 414.095 | 5.5E-03 | NEG | [M-H]- | 346 | 273780 | 5.1 |
| 1.0 | 414.226 | 2.2E-18 | NEG | [M-H]- | 142 | 47219 | 17.7 |
| 5.1 | 415.097 | 6.9E-03 | NEG | [M-H]- | 27 | 54654 | 2.8 |
| 5.2 | 415.098 | 4.7E-03 | NEG | [M-H]- | 81 | 55541 | 8.9 |
| 5.0 | 415.177 | 3.8E-02 | NEG | [M-H]- | 54 | 22127 | 18.6 |
| 5.1 | 415.178 | 4.9E-02 | NEG | [M-H]- | 33 | 19755 | 28.6 |
| 1.0 | 415.236 | 1.3E-07 | NEG | [M-H]- | 13 | 17330 | 8.2 |
| 5.2 | 416.092 | 8.8E-03 | NEG | [M-H]- | 143 | 98811 | 7.0 |
| 5.1 | 416.093 | 8.6E-03 | NEG | [M-H]- | 135 | 98532 | 3.4 |
| 7.4 | 417.233 | 3.2E-02 | NEG | [M-H]- | 35 | 21615 | 28.9 |
| 4.9 | 427.178 | 3.6E-02 | NEG | [M-H]- | 12 | 7308 | 20.5 |
| 0.9 | 434.024 | 4.4E-02 | NEG | [M-H]- | 30 | 18506 | 26.2 |
| 4.7 | 448.304 | 3.8E-02 | NEG | [M-H]- | 153 | 169655 | 6.6 |
| 1.4 | 450.204 | 2.3E-02 | NEG | [M-H]- | 11 | 5706 | 10.6 |
| 1.3 | 450.205 | 2.6E-02 | NEG | [M-H]- | 10 | 6620 | 21.8 |
| 3.2 | 459.194 | 3.6E-02 | NEG | [M-H]- | 61 | 37221 | 29.4 |
| 0.8 | 466.218 | 6.3E-14 | NEG | [M-H]- | 13 | 3760 | 18.2 |
| 5.2 | 471.240 | 3.3E-02 | NEG | [M-H]- | 75 | 16367 | 7.9 |
| 5.1 | 471.243 | 7.4E-03 | NEG | [M-H]- | 40 | 48233 | 17.3 |
| 5.1 | 471.250 | 7.0E-03 | NEG | [M-H]- | 29 | 33598 | 28.7 |
| 6.6 | 476.275 | 4.4E-02 | NEG | [M-H]- | 1123 | 468678 | 1.7 |
| 8.5 | 482.928 | 3.7E-02 | NEG | [M-H]- | 11 | 8695 | 7.0 |
| 6.5 | 500.277 | 4.4E-02 | NEG | [M-H]- | 2647 | 2741210 | 14.9 |
| 7.1 | 500.618 | 4.2E-02 | NEG | [M-H]- | 12 | 8389 | 12.4 |
| 6.0 | 507.270 | 4.9E-02 | NEG | [M-H]- | 633 | 641299 | 11.3 |
| 6.8 | 514.283 | 3.7E-02 | NEG | [M-H]- | 4771 | 3812673 | 7.4 |
| 3.1 | 523.263 | 4.1E-02 | NEG | [M-H]- | 35 | 30484 | 27.3 |
| 6.5 | 526.289 | 9.8E-03 | NEG | [M-H]- | 1191 | 679292 | 17.6 |
| 6.5 | 526.291 | 2.5E-02 | NEG | [M-H]- | 661 | 640122 | 4.1 |
| 3.0 | 530.966 | 2.2E-02 | NEG | [M-H]- | 10 | 383352 | 14.3 |
| 6.5 | 534.351 | 4.4E-02 | NEG | [M-H]- | 17 | 5487 | 14.7 |
| 3.1 | 545.245 | 1.3E-02 | NEG | [M-H]- | 16 | 16350 | 20.4 |
| 6.2 | 549.158 | 2.2E-02 | NEG | [M-H]- | 25 | 10516 | 14.4 |
| 5.9 | 551.098 | 4.3E-02 | NEG | [M-H]- | 13 | 12459 | 12.7 |
| 6.4 | 552.306 | 4.6E-02 | NEG | [M-H]- | 859 | 376771 | 17.9 |
| 6.5 | 560.485 | 3.9E-02 | NEG | [M-H]- | 38 | 25460 | 11.2 |
| 6.2 | 594.211 | 3.5E-02 | NEG | [M-H]- | 17 | 8893 | 18.0 |
| 6.3 | 599.238 | 4.6E-02 | NEG | [M-H]- | 69 | 23445 | 13.1 |
| 5.2 | 606.120 | 2.4E-03 | NEG | [M-H]- | 11 | 3779 | 15.0 |
| 6.8 | 607.944 | 4.7E-02 | NEG | [M-H]- | 40 | 33802 | 9.6 |
| 6.6 | 620.244 | 8.7E-03 | NEG | [M-H]- | 11 | 4960 | 14.7 |
| 6.6 | 626.136 | 1.1E-02 | NEG | [M-H]- | 16 | 14268 | 10.6 |
| 6.5 | 626.429 | 2.4E-02 | NEG | [M-H]- | 18 | 9915 | 14.8 |
| 6.5 | 638.415 | 4.1E-02 | NEG | [M-H]- | 13 | 7907 | 22.0 |
| 5.9 | 642.306 | 1.6E-02 | NEG | [M-H]- | 17 | 4637 | 8.6 |
| 6.2 | 656.367 | 3.2E-02 | NEG | [M-H]- | 16 | 14423 | 14.0 |
| 7.7 | 663.271 | 4.6E-02 | NEG | [M-H]- | 74 | 1100 | 18.1 |
| 6.8 | 664.143 | 4.6E-02 | NEG | [M-H]- | 85 | 33438 | 12.2 |
| 6.4 | 672.290 | 2.6E-02 | NEG | [M-H]- | 23 | 15594 | 25.3 |
| 6.6 | 676.145 | 1.2E-02 | NEG | [M-H]- | 26 | 24474 | 15.5 |
| 6.6 | 676.147 | 1.4E-02 | NEG | [M-H]- | 32 | 25586 | 15.5 |
| 9.0 | 685.348 | 3.8E-02 | NEG | [M-H]- | 20 | 1291 | 17.9 |
| 5.8 | 691.201 | 4.5E-02 | NEG | [M-H]- | 33 | 33423 | 20.6 |
| 6.6 | 700.066 | 4.8E-02 | NEG | [M-H]- | 12 | 12218 | 6.5 |
| 6.6 | 700.067 | 2.6E-02 | NEG | [M-H]- | 18 | 12799 | 8.8 |
| 6.5 | 714.383 | 4.0E-02 | NEG | [M-H]- | 21 | 13538 | 17.9 |
| 6.6 | 716.219 | 4.5E-02 | NEG | [M-H]- | 28 | 21216 | 9.7 |
| 5.7 | 736.488 | 1.0E-02 | NEG | [M-H]- | 1603 | 479098 | 8.9 |
| 5.7 | 736.488 | 7.4E-03 | NEG | [M-H]- | 489 | 478848 | 9.0 |
| 8.8 | 740.389 | 2.0E-02 | NEG | [M-H]- | 16 | 3889 | 17.8 |
| 6.5 | 747.310 | 3.8E-02 | NEG | [M-H]- | 20 | 7594 | 17.5 |
| 9.1 | 751.394 | 2.4E-02 | NEG | [M-H]- | 24 | 1141 | 18.5 |
| 5.9 | 756.510 | 3.8E-02 | NEG | [M-H]- | 14 | 13896 | 8.2 |
| 5.6 | 760.486 | 4.9E-02 | NEG | [M-H]- | 190 | 239562 | 8.6 |
| 6.4 | 762.208 | 4.2E-02 | NEG | [M-H]- | 27 | 7918 | 24.6 |
| 5.0 | 767.484 | 1.6E-02 | NEG | [M-H]- | 25 | 10812 | 12.6 |
| 6.6 | 768.120 | 3.4E-02 | NEG | [M-H]- | 11 | 6291 | 24.0 |
| 6.5 | 780.290 | 2.0E-02 | NEG | [M-H]- | 38 | 18206 | 13.0 |
| 5.9 | 782.525 | 4.4E-02 | NEG | [M-H]- | 30 | 28261 | 14.3 |
| 5.8 | 782.526 | 1.3E-02 | NEG | [M-H]- | 30 | 27710 | 10.4 |
| 5.0 | 793.499 | 2.1E-02 | NEG | [M-H]- | 24 | 23731 | 20.4 |
| 5.1 | 795.514 | 4.9E-02 | NEG | [M-H]- | 29 | 34565 | 11.5 |
| 5.7 | 816.564 | 4.8E-02 | NEG | [M-H]- | 23 | 13646 | 28.2 |
| 0.8 | 819.452 | 4.5E-09 | NEG | [M-H]- | 31 | 1285 | 16.7 |
| 6.6 | 852.029 | 4.6E-02 | NEG | [M-H]- | 48 | 35926 | 19.4 |
| 6.5 | 858.209 | 2.0E-02 | NEG | [M-H]- | 11 | 5078 | 22.8 |
| 6.9 | 866.527 | 4.8E-02 | NEG | [M-H]- | 12 | 10402 | 28.0 |
| 5.9 | 866.567 | 3.8E-02 | NEG | [M-H]- | 28 | 22059 | 29.2 |
| 5.9 | 866.570 | 2.8E-02 | NEG | [M-H]- | 22 | 22154 | 28.3 |
| 6.1 | 878.584 | 3.2E-02 | NEG | [M-H]- | 54 | 62689 | 13.5 |
| 3.0 | 894.943 | 2.7E-02 | NEG | [M-H]- | 20 | 37877 | 26.8 |
| 6.5 | 902.029 | 4.3E-02 | NEG | [M-H]- | 10 | 5553 | 14.8 |
| 5.7 | 980.555 | 4.4E-02 | NEG | [M-H]- | 39 | 33846 | 12.3 |
| 5.7 | 980.558 | 4.8E-02 | NEG | [M-H]- | 56 | 33708 | 11.2 |

**
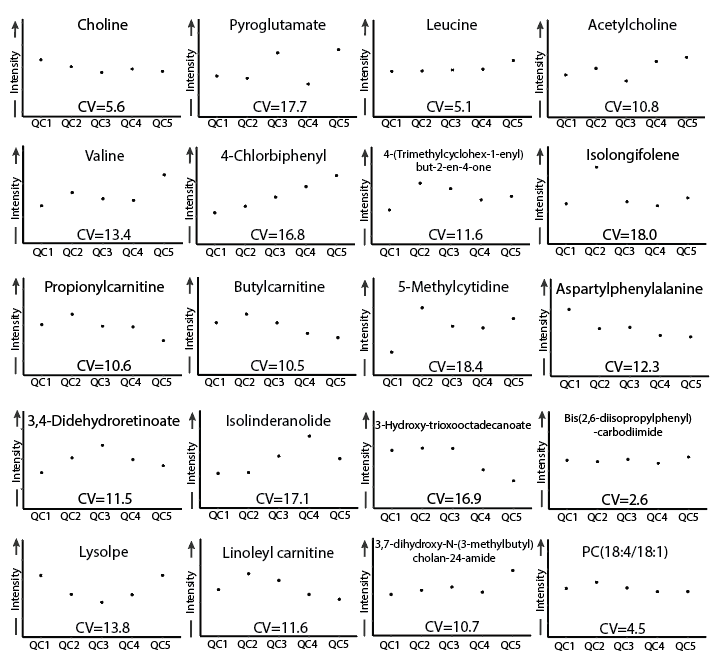
**

**Supplemental Fig. 1 Plots of relative concentration in QC samples for selected annotated features** Plots were created with data from pooled quality control samples to determine reproducibility across the analytical batch.


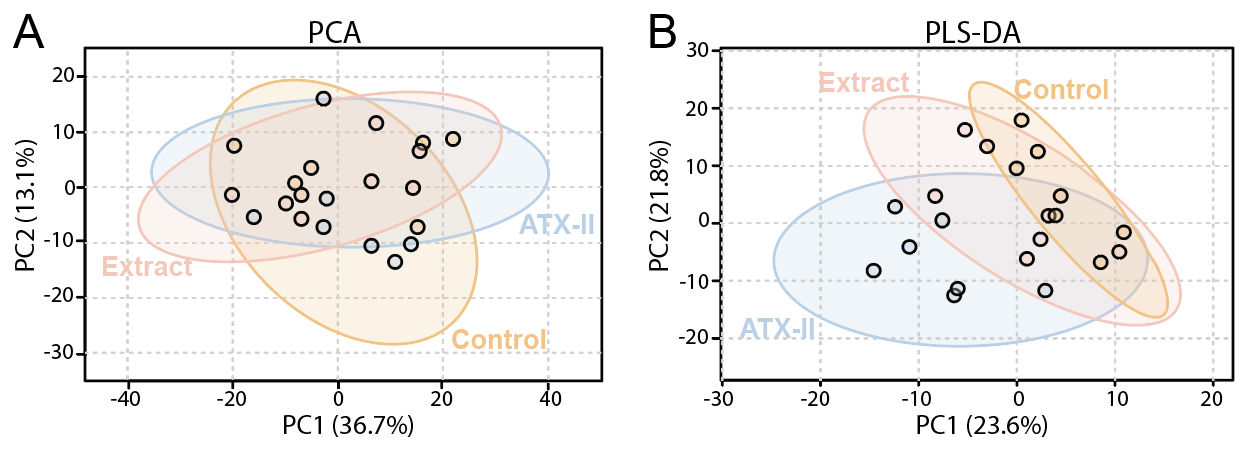


**Supplemental Fig. 2 Global untargeted statistical analysis of data collected in (-) mode** (a) PCA showing similar metabolomic profiles in the (-) dataset. (b) PLS-DA indicating some differences between the three groups. Separation is seen between ATX-II and the cultured extract toxin mixture relative to the control. This trend is highlighted by an increased disparity between ATX-II and the control relative to the cultured extract complex mixture and the control.


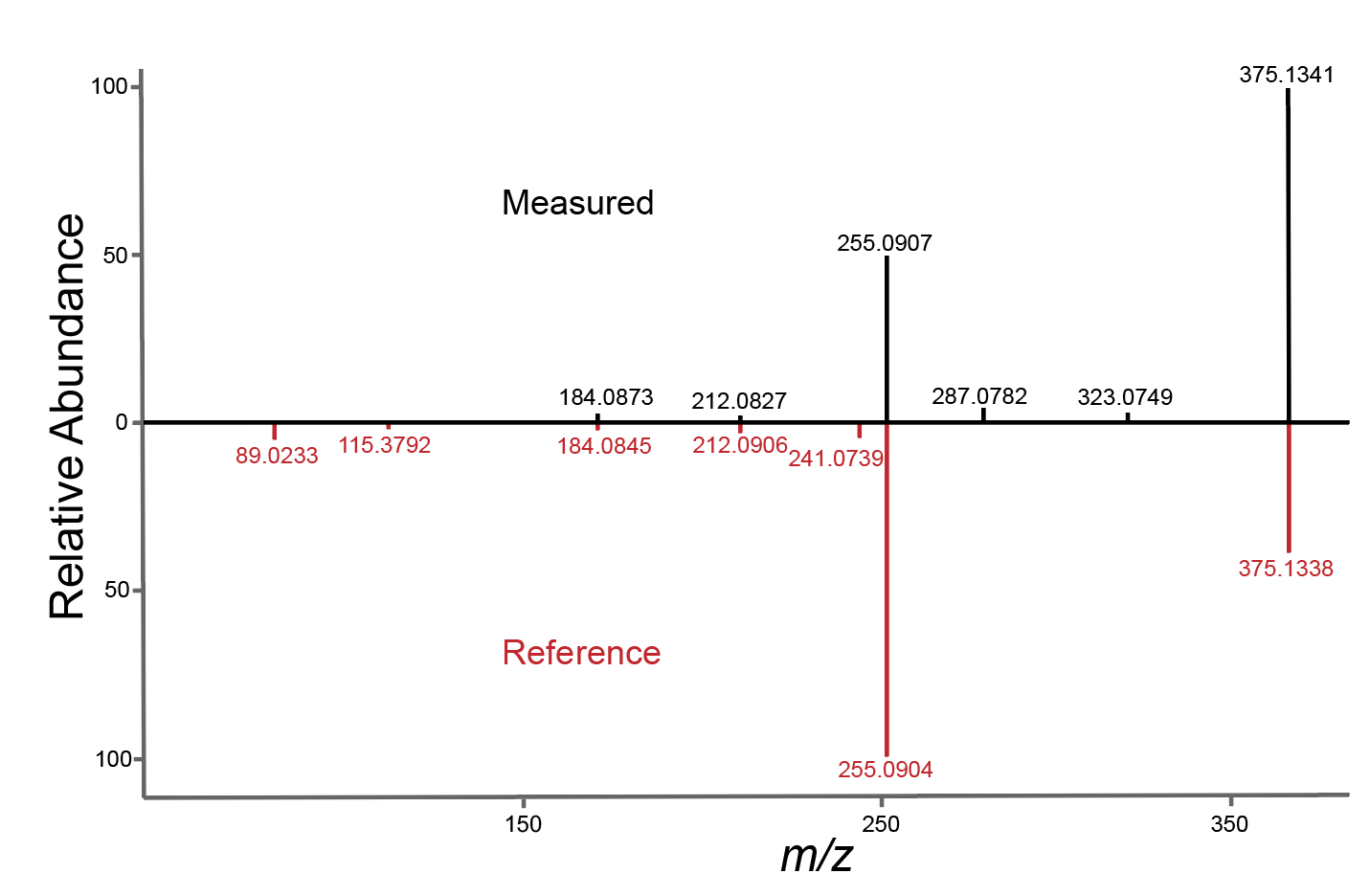


**Supplemental Fig. 3 Riboflavin identification** Reference standard spectra comparison of riboflavin vs measured spectra in rat livers annotation as riboflavin.


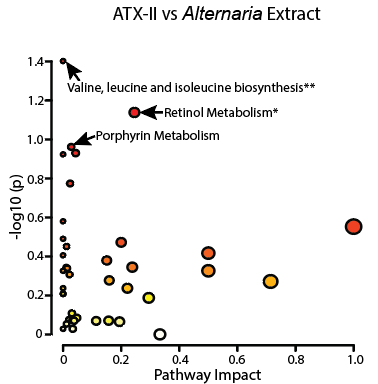


**Supplemental Fig. 4 Pathway analysis comparison between rats receiving ATX-II and the complex toxin mixture** Valine, leucine and isoleucine metabolism was significantly disrupted between the two toxin doses. Retinol metabolism was found to be slightly disrupted. Significance and pathway impact are plotted on the y and x axis, respectively. The size of the dot is indicative of the pathway impact while red colors indicate higher levels of significance. Pathways marked with an * have *P-*values <0.1, ** denoted pathways have *P-*values <0.05 and *** indicated pathways have *P-*values >0.01.


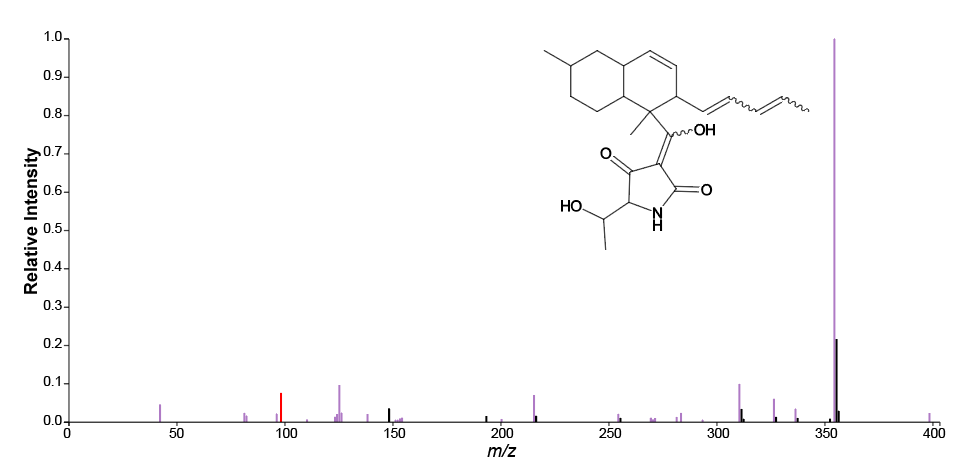


**Supplemental Fig. 5 Annotation of altersetin** *In-silico* structural annotation using SIRIUS software and collected fragmentation data. Predicted fragmentation matches in purple correspond to structural components of altersetin.

**
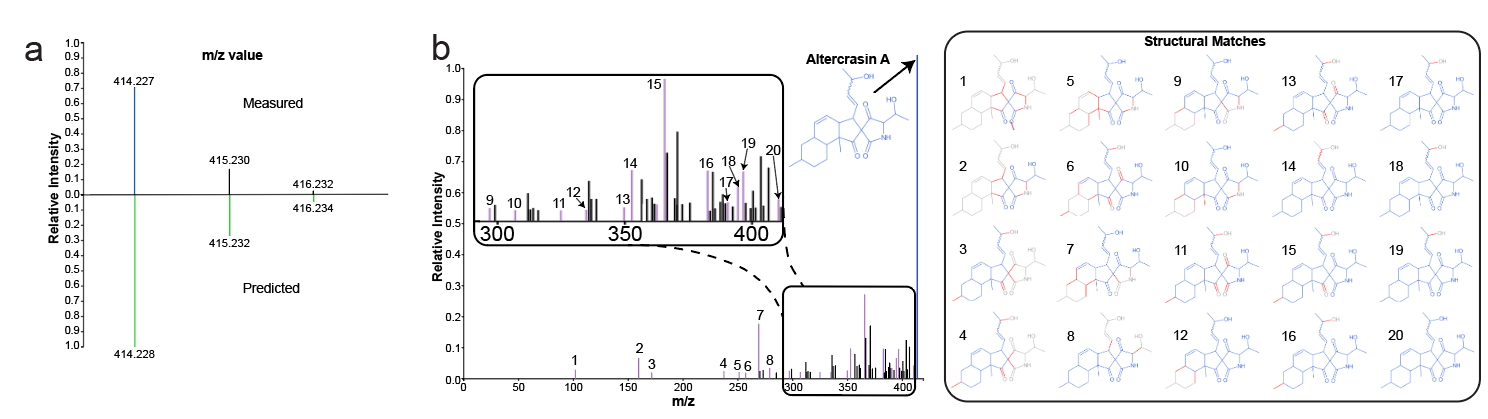
**

**Supplemental Fig. 6 Annotation of altercrasin A** (a) Isotopic distribution of the acquired data in blue for the m/z value associated with altercrasin A and the predicted distribution in green. (b) In-sillico structural annotation using SIRIUS software and collected fragmentation data. Predicted fragmentation matches in purple on the left are indicated by numbers which correspond to structural components of altercrasin A on the right.
